# Graviola Extract versus Adipose-Derived Mesenchymal Stem Cells as Therapeutics in Repairing Liver Damage Caused by 2‑Amino-3-Methylimidazo[4, 5‑f]quinoline

**DOI:** 10.1021/acsomega.5c03088

**Published:** 2025-11-05

**Authors:** Doaa Hamada Abd El-Hafeez Thabet, Mona M. Atia, Hanem S. Abdel-Tawab, Alshaimaa A. I. Alghriany

**Affiliations:** Laboratory of Molecular Cell Biology, Zoology and Entomology Department, Faculty of Science, 200940 Assiut University, Assiut 71516, Egypt

## Abstract

The mutagenic and carcinogenic heterocyclic amine, 2-amino-3-methylimidazo­[4,5-f]­quinoline (IQ), is produced while cooking protein-rich foods. Mesenchymal stem cells (MSCs) (as cell-based therapy) and *Annona muricata* (graviola) (as a natural product) both possess preventive capacities against free radical toxicity in various tissues. This study aims to compare the therapeutic properties and effects of AD-MSCs and graviola on IQ-induced liver toxicity and DNA damage in rats. Sixty adult male rats were divided into four groups: normal unexposed control, IQ, IQ + graviola, and IQ + AD-MSCs. After 6 weeks, the rats were sacrificed, and liver tissues were examined for histopathological changes using hematoxylin–eosin staining. p53 protein expression was evaluated by immunohistochemistry, and DNA damage was measured by using the comet test. Our findings indicated that AD-MSC therapy led to the most significant improvement in DNA damage, apoptosis, and p53, LPO, AST, and ALT levels caused by IQ toxicity. Additionally, AD-MSCs reduced severe histological alterations (damage and fibrosis) in liver cells induced by IQ. However, the effectiveness of graviola treatment is limited, severely restricting its use for chronic liver toxicity. In conclusion, the initial stage of IQ-induced liver toxicity is caused by oxidative stress-induced DNA damage. Compared with graviola, AD-MSCs exhibit more potent therapeutic effects against IQ-induced liver damage.

## Graphical abstract

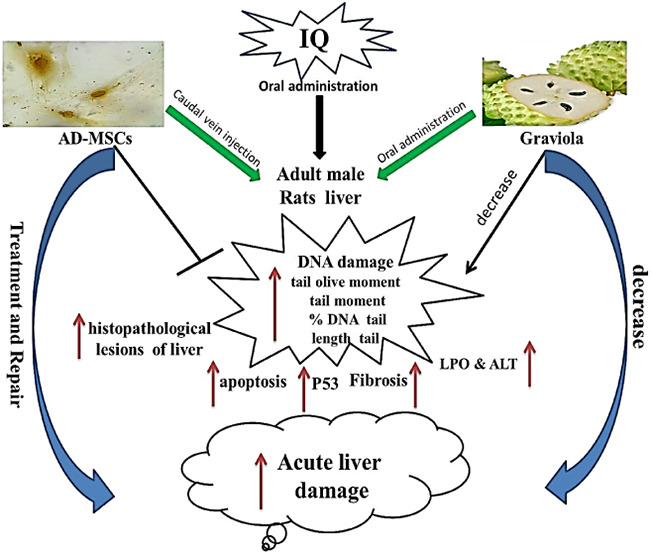

## 1 Introduction

The recognized heterocyclic compound 2-amino-3-methylimidazo­[4,5-f]­quinolone (IQ) exhibits genotoxic properties when subjected to elevated cooking temperatures, especially in protein-rich foods like meat and fish.^1^ Consequently, even modest dosages of IQ may exhibit carcinogenic properties, despite the low amounts of IQ present in food.^2,3^ IQ and HCAs are linked to liver, lung, brain, forestomach, colon,^4,5^ mammary, and Zymbal gland cancers in mice, rats, and zebrafish.^6,7^

Through the MAPK and NF-κB signaling pathways, IQ causes oxidative stress and inflammation in zebrafish (*Danio rerio*), leading to hepatotoxic effects.^8^ Worries about dietary and environmental exposure dangers are anticipated to continue due to IQ’s toxicity to organisms.^9^ This concern arises from the theory that genotoxic carcinogens, even at minimal concentrations, can permanently modify the genetic composition of target organ cells. Furthermore, the mechanisms underlying the carcinogenicity of lower doses of HCA remain unknown.^2^ Possible signs of oxidative stress include the presence of markers associated with nucleic acid damage.^10^

In recent years, fresh fruits have become increasingly popular because of their inherent antioxidant capabilities. Similarly, utilization of stem cells derived from human adipose tissue in the context of cell therapy has become increasingly common.^11^ Mesenchymal stem cells (MSCs) are utilized in regenerative medicine because they proliferate and differentiate into various cell types. Canine adipose-derived stem cells (ADSCs) have been used for treating immune-mediated diseases in dogs^12–14^ and inflammatory bowel disease (IBD).^15^ However, human adipose-derived stem cells, or ASCs, have anti-inflammatory, immunomodulatory, and antioxidant characteristics.^16–18^

Furthermore, in long-term culture, AD-SCs showed increased genetic and morphological stability.^19,20^ ADSCs are especially well-suited for cell-based therapy.^21^ Specifically, ADSC transplantation has shown promising results in treating liver cirrhosis in preclinical studies^22,23^ and nonalcoholic liver diseases,^24^ all in a short period.^25^

Research has shown that natural medicines have long been used to treat various illnesses.^26^ Graviola (*Annona muricata*) fruits contain an excessive amount of water, carbohydrates, salts, and vitamins, which makes their flesh and pulp perfect for preparing juices and other drinks.^11^ The seed, fruit pulp, and leaves of *Annona muricata* extracts contain a wide range of bioactive compounds, including many tannins, coumarins, aporphine and isoquinoline alkaloids, megastigmanes, and acetogenins.^27–29^

Several studies have indicated that these organic compounds possess antioxidant qualities.^30,31^ Additionally, graviola has shown potential antidiabetic and antilipogenic effects,^32^ along with antiviral properties.^33^ Graviola extracts have been found to contain flavonoid classes, phenolic content, and acetogenins, all of which exhibit significant radical scavenging activity. The radical scavenging activity of the extracts is positively correlated with the overall concentration of these compounds.^34,35^ Both in vitro and in vivo studies have revealed that these compounds exhibit a wide range of biological and therapeutic actions.^36^

Little is known about IQ-induced liver toxicity regarding liver damage in comparison to other organs. This study assessed the correlation between low-dose IQ administration and liver damage by evaluating oxidative DNA damage, apoptosis, and p53 levels. Furthermore, the study compared the effects and therapeutic properties of administering AD-MSC and graviola for long-term liver damage induced by IQ in rats.

## 2 Materials and Methods

### 2.1 Materials

IQ was sourced from Sigma-Aldrich, GmbH, Munich, Germany, while graviola was obtained from a commercial supermarket. The primary antibody α p53 Mo (1:10,000) and secondary antibody Rb α Mo were bought from Sigma Co. (St. Louis, MO). An RPMI growth medium (with l-glutamine), fetal bovine serum (FBS), and an antibiotic mix (10,000 U penicillin/ml and 10,000 U streptomycin/ml) were obtained from Gibco (Invitrogen, CA, USA). Collagenase type II was sourced from Sigma-Aldrich, St. Louis, MO, USA. Mouse primary anti-CD105 and CD90 antibodies and IgG and anti-CD45 antibodies were purchased from Thermo Fisher. Ultra-Tek polyvalent goat antimouse HRP was purchased from Sky Tek Laboratories, Logan, Utah, USA.

### 2.2 Ethical Approval

In accordance with the National Institutes of Health rules, the research procedures used in this work were examined and approved by Assiut University’s Faculty of Science Research Ethics Committee (FSREC) (IRB No: 01–2024–0004).

### 2.3 Experimental Design

Adult male rats (*n* = 60, 10 rats per group) weighing 120–150 g were randomized and divided into four groups. Group 1: controls, divided into (A) no treatment; (B) 0.1 mL of 70% ethanol; and (C) 0.2 mL of corn oil. Group 2: IQ 50 mg/kg body weight, dissolved in 0.2 mL of corn oil for 30 days.^37^ Group 3: IQ + graviola 10 mg/kg body weight,^38^ oral administered for 30 days of IQ, followed by graviola for 15 days. Rats were sacrificed after the 45-day period. Group 4: IQ + AD-MSCs, with each rat injected into the caudal vein with 0.65 × 10^6^ AD-MSCs in 0.5 mL of PBS^39^ after 30 days of IQ administration. AD-MSCs were administered for 15 days, and rats were sacrificed after the 45-day period. Rats were anaesthetised via intraperitoneal injection of ketamine and xylazine and subsequently sacrificed by slaughtering.

### 2.4 Preparations of IQ

It was weighed and subsequently dissolved in distilled water immediately prior to use.

### 2.5 Graviola Extraction

Five entire soursops (*Annona muricata* L.) fruits, weighing between 0.5 and 2.0 kg, with slightly ripened yellowish-green peels, were randomly selected from a commercial supermarket in Egypt. The fruits were washed and cut into small pieces, and the pulps, peels, and seeds were cut into uniform pieces (1 cm^3^). The powdered soursop fruit pulps, peels, and seeds (5 g) were extracted using 50 mL of 100% ethanol and shaken in a shaking incubator at 150 rpm for 1 h at room temperature (25 °C). The residues were collected, re-extracted with ethanol, and centrifuged for 15 min at 4500 rpm. The supernatant was concentrated in a rotary evaporator set at 40 °C and then oven-dried at 37 °C for 24 h until a constant weight was obtained, producing a powder from the soursop meat and peel. The powder was stored at −20 °C for further study.^40,41^

#### 2.5.1 GC–MS Analysis for Graviola Extraction

GC–MS (7890–5975) analysis was conducted using helium as the carrier gas. A DB-5MS column (5% phenyl methyl siloxane, 30 m × 0.25 mm × 0.25 μm) combined with a mass-selective detector (GCMS-QP2010 Ultra, Shimadzu, Kyoto, Japan) was used. The carrier gas flow rate was 1.0 mL/min. The GC/MS conditioning included an oven equilibration time of 0.5 min, with a maximum initial column temperature of 220 °C (1 min after injection). The oven program was as follows: 40 °C for 2 min, then increased by 10 °C/min to 150 °C for 6 min, increased by 10 °C/min to 220 °C for 6 min, and increased by 15 °C/min to 280 °C for 15 min, totaling 51 min, plus 48 min, and 2 min postrun at 260 °C. After a 1 min holding period, the temperature was raised to 220 °C with an 8 °C/min heating ramp and then to 280 °C with a 2 °C/min heating ramp in 15 min.^42^ Mode, spitless; Flow program, 0.5 mL/min for 10.9 min then 1 mL/min per min to 1 mL/min for 30. MS Source: 230 °C to max. 250 °C; MS Quad: 150 °C to max. 200 °C. Components were identified by comparing their mass spectra with those in the Wiley 9 and NIST libraries. The computerized integrator extracted the relative percentages of isolated substances from the total ion chromatogram at the Analytical Chemistry Unit (ACAL) in Assiut University.

### 2.6 AD-MSC Isolation and Culture

After being extracted from the visceral lipids of adult rats, the fats were cut into 1–3 mm pieces. To remove any remaining blood, the adipose tissues were washed three times with a sterile phosphate buffer solution. The adipose tissue pieces were then enzymatically broken down using 0.25% collagenase type II in PBS with 20% fetal bovine serum (FBS) for 45–60 min at 37 °C, with 15 min shaking intervals. The activity of the collagenase was reduced by adding 5 mL of FBS. Following centrifugation, the pellets were filtered through a 40 μm cell strainer and suspended in 12 mL of Dulbecco’s Modified Eagle’s Medium (DMEM) culture media. The supernatant was removed after 10 min of centrifugation at 1800 rpm. The cell suspension was then placed in a culture flask and incubated at 37 °C and 5% CO_2_. AD-MSCs at passage 3 (P3) were deemed suitable for transplantation after approximately 15 days of growth, reaching roughly 80% confluency.^38,39,43^

### 2.7 Immunocytochemistry Method for Characterization of AD-MSCs

After being incubated with 4% paraformaldehyde for 20 min at room temperature, the cells were washed in PBS. The cells were permit for 5 min with fresh 0.2% Triton X-100 in PBS, followed by another PBS wash.^44^ After that, the slides were incubated for 10 min in a blocking buffer. The cells were treated with the primary antibodies CD105, CD90 (2:100), and CD45 (1:100) for one h at room temperature. The cells underwent four PBS washes, following incubation. As instructed by the manufacturer, the Ultra-Tek antipolyvalent stain was applied, and it was then allowed to soak at room temperature for 10 min. The slides were then analyzed as soon as the DAB substrate mixture and DAB chromogen were mixed.

### 2.8 Comet Assay Detection

For slide coating, 200 μL of cell suspension was combined with 800 μL of low-melting agarose (0.8% in PBS). The coated slides were submerged in a lysis buffer comprising 0.045 M Tris/borate/EDTA buffer (TBE), pH 8.4, and 2.5% sodium dodecyl sulfate (SDS) for 15 min. In an electrophoresis chamber with TBE buffer devoid of SDS, the slides were placed. Electrophoresis was carried out for two min at 100 mA and 2 V/cm. At 4 °C, 20 μg/mL of ethidium bromide (EtBr) was used to stain the slides. A fluorescent microscope with a 40× objective and an excitation filter of 420–490 nm was used to detect DNA damage.^45^ The OpenComet software was used to evaluate tail length, tail moment, olive tail moment, and the percentage of DNA in the head from 5 images, allowing both qualitative and quantitative assessments of DNA damage.

### 2.9 Immunohistochemistry Detection

Paraffin-embedded tissues were deparaffinized using xylene and subsequently rehydrated through a sequence of ethanol solutions. Antigen retrieval was conducted by boiling the slides in 1 mM EDTA for 10 min. The sections were subsequently treated with 3% H_2_O_2_ for 5 min to inhibit endogenous peroxidase activity, rinsed with 1× PBS for 5 min, and subjected to blocking for 1 h at room temperature. A 1:1000 dilution of the p53 primary antibody was subsequently introduced. Subsequent to the removal of the antibody solution, the sections were rinsed with a wash buffer for 10 min. Secondary antibodies at a 1:5000 dilution were applied and incubated for 30 min before removal. The sections were rewashed and stained with counterstain and 3,3′-diaminobenzidine (DAB) for 2 to 3 min.^46^

### 2.10 Apoptosis Detection by Using Acridine Orange Staining

Acridine orange (AO), when exposed to blue light in its monomeric form, displays metachromatic properties and emits green fluorescence under a microscope at an excitation–emission wavelength of approximately 488 nm. It produces green fluorescence upon binding to DNA. The sample was quickly passed through a series of alcohol concentrations (80%, 70%, and 50%), distilled water, and then immersed in a 0.01% AO staining solution (prepared by diluting a 0.1% AO stock solution in distilled water using phosphate buffer to reach a pH of 7.2). After staining, the sample was transferred into PBS for 1 min, differentiated in 0.10 M CaCl_2_ for 2 min, immediately rinsed with phosphate buffer, and mounted wet with a cover glass for inspection.^47,48^

### 2.11 Liver Function Detection and LPO Detection for Oxidative Stress

Liver function tests for alanine aminotransferase (ALT) and aspartate aminotransferase (AST) activity in blood plasma were performed using a kit from Spectrum Diagnostics company in Egypt, following the manufacturer’s procedures. According to a study,^49^ malondialdehyde was used as thiobarbituric acid to evaluate lipid peroxidation (LPO) in the liver. After homogenization, 1% (v/v) DMSO was added to prevent further oxidation. Tissue homogenates in 0.2 mL aliquots were combined with the reaction buffer and subjected to spectrophotometric measurement.

### 2.12 Histological and Histopathological Examination

For general histological examination, the standard staining procedure using hematoxylin and eosin stain was performed.^50^ Nine liver tissue lesions were evaluated histopathologically. The outcomes have been grouped into four categories: (−) absent lesion, (+) slight (<25%), (++) moderate (from 25 to 50%), and (+++) severe (>50%).^51^ Picro-Sirius red stain was used for collagen identification.^51^ A digital camera (ToupTek ToupView, Copyright 2019, Version X86, Compatible: Windows XP/Vista/7/8/10, China), ImageJ software, and a computer connected to a light microscope (Olympus CX31, Japan) were used for examination and photography.

### 2.13 Statistical Analysis

The data were expressed as mean ± SE. The Student’s *t* test was employed to compare parameters between two groups, whereas multiple comparisons, performed in a minimum of three separate determinations, were assessed using one-way analysis of variance (ANOVA). Statistical significance was considered at *p* < 0.001 and *p* < 0.05. Data analysis was conducted utilizing Prism software version 8.4.3 (686), Fiji/ImageJ, and OpenComet for graphical representation. Photography was conducted using a digital camera (ToupTek ToupView, Copyright 2019, Version: x86, Compatible: Windows XP/Vista/7/8/10, China).

## 3 Results

### 3.1 GC/MS Analysis Identification and Quantification of the Chemical Constituents

Figure 1 shows the chromatograms of GC–MS spectral analysis, while Table 1 presents 85 peaks of compounds extracted from graviola (pulps, peels, and seeds) of the *Annona muricata* fruit. The main components identified include urethylane (10.97%), erythritol (7.22%), glycolic acid (7.18%), 4-mercaptophenol (6.73%), and 1,3-dihydroxy-2-propanone (5.93%). Identification was based on mass spectral data, including molecular ion peaks, fragmentation patterns, and Kovats retention index. The GC–MS complete analysis and all data are in Figures S1, S2 and Table S1.

**Figure 1:**
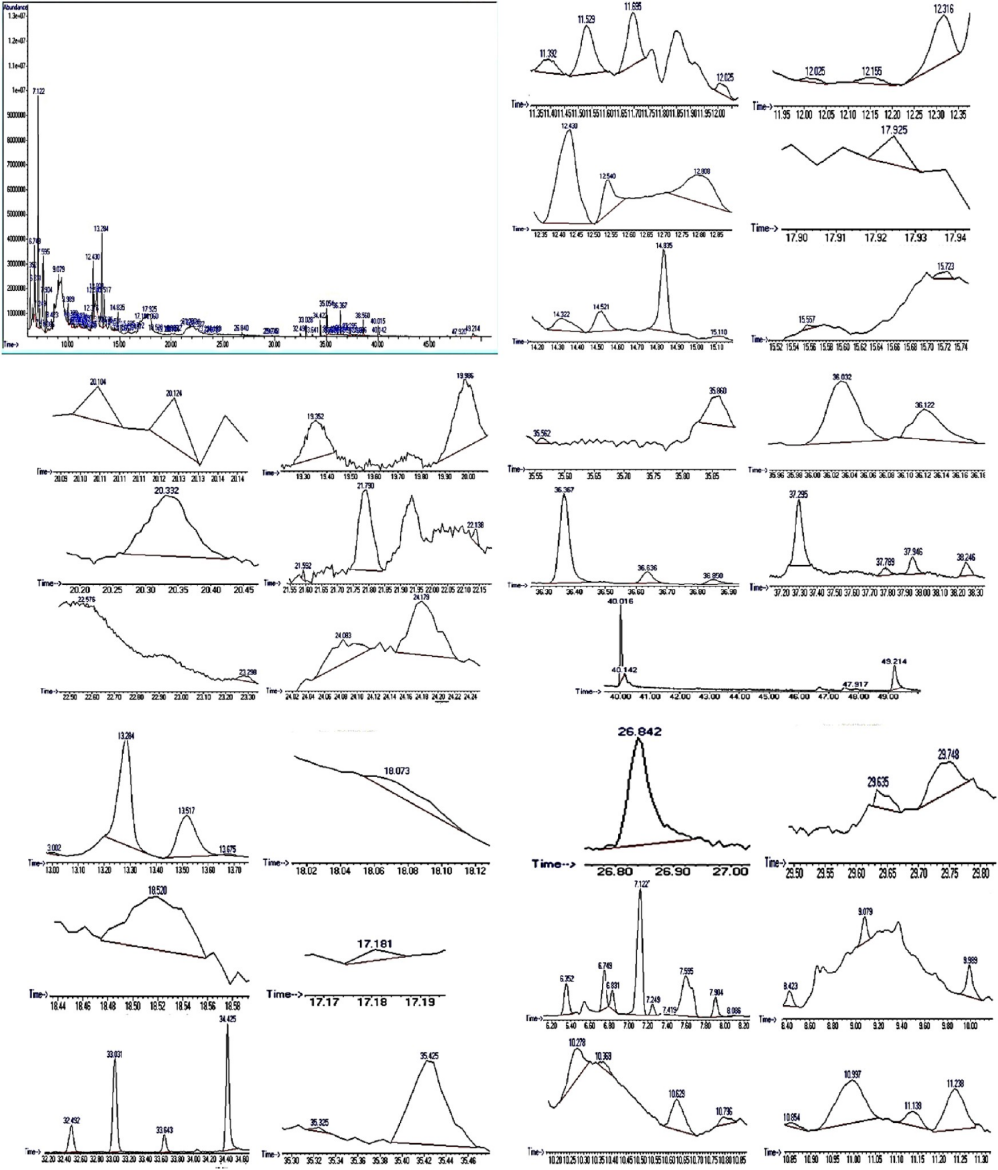
Chromatogram of GC–MS spectral analysis; the identification was made using the mass spectral data (molecular ion peaks, fragmentation patterns).

**Table 1:** Chemical Compounds of Graviola (*A. muricata* L.) (Pulps, Peels, and Seeds) Extract Identified by GC–MS

Analyte/Parameter	Test Method	Description
Glutaric Acid, Monophenyl Ester	ACAL-APR-01-00	Value: 0.168% Retention time: 32.492 min
1,2-Diformoxyethane	ACAL-APR-37-00	Value: 0.585% Retention time: 10.803 min
Carbonic Acid, Diethyl Ester	ACAL-APR-37-00	Value: 0.251% Retention time: 10.854 min
1-Piperidinoacetonitrile	ACAL-APR-37-00	Value: 0.086% Retention time: 33.643 min
2-(2-Aminopropyl)phenol	ACAL-APR-37-00	Value: 0.019% Retention time: 29.639 min
2,4-Diamino-1,3, 5-Triazin-6-One	ACAL-APR-37-00	Value:0.041% Retention time: 20.124 min
2-Propoxyethanol	ACAL-APR-37-00	Value: 0.082% Retention time: 19.354 min
3-Hydroxy-2(5h)-Furanone	ACAL-APR-37-00	Value: 0.152% Retention time: 20.337 min
2-Hydroxyethyl Thiadiazol-2-Yl Amine	ACAL-APR-37-00	Value: 0.432% Retention time: 21.592 min
4-Ethyl-5-[[2-[5-[(3-Ethyl-1,5-Dihydro-4-Methyl- 5-Oxo-2h-Pyrrol-2-Ylidene)Methyl]-3,4-Dimethy l-2h-Pyrrol-2-Ylidene]-3,4-Dimethyl-2h-Pyrrol-5- Yl]Methylene]-1,5-Dihydro-3-Methyl-, (E,Z,Z)-2h-Pyrrol-2-One	ACAL-APR-37-00	Value: 0.059% Retention time: 29.749 min
4-Fluoro-3-[1-hydroxy-2-(methylamino)ethyl] phenol	ACAL-APR-37-00	Value: 0.017% Retention time: 37.79 min
5-Amino-1H-Pyrazole-4-carbothioamide	ACAL-APR-37-00	Value: 1.719% Retention time: 12.543 min
3-Amino-2-oxazolidone	ACAL-APR-37-00	Value: 0.428% Retention time: 11.139 min
2-Isopropylpiperazine	ACAL-APR-37-00	Value: 0.082% Retention time: 12.155 min
1,3-Dihydroxy-2-Propanone	ACAL-APR-37-00	Value: 5.931% Retention time: 9.082 min
(−)-Adrenaline	ACAL-APR-37-00	Value: 0.043% Retention time: 24.083 min
1,4:3,6-Dianhydro-, Dinitrate d-Glucitol	ACAL-APR-37-00	Value: 0.530% Retention time: 11.398 min
1,4-Anhydro-d-mannitol	ACAL-APR-37-00	Value: 0.055% Retention time: 16.489 min
1,4-Diacetyl-3-acetoxymethyl-2,5-methylene-l-r hamnitol	ACAL-APR-37-00	Value: 0.020% Retention time: 20.104 min
1,5-Bis(3,4-Dihydroxyphenyl)Pentane	ACAL-APR-37-00	Value: 0.530% Retention time: 34.426 min
1,6-Anhydro-2,4-dideoxy-beta-d-ribo-hexopyranose	ACAL-APR-37-00	Value: 0.162% Retention time: 18.52 min
12-Methylaminolauric acid	ACAL-APR-37-00	Value: 0.011% Retention time: 47.92 min
17-(Acetyloxy)-, (4.beta)-Kauran-18-al	ACAL-APR-37-00	Value: 0.636% Retention time: 38.56 min
1-Butyl-2-oxo-1-propylhydrazine	ACAL-APR-37-00	Value: 0.638% Retention time: 14.522 min
1-Deoxy-d-mannitol	ACAL-APR-37-00	Value: 0.206% Retention time: 22.142 min
1-Nitro-2-acetamido-1,2-dideoxy-d-monnitol	ACAL-APR-37-00	Value: 0.128% Retention time: 16.715 min
2,4-Dihydroxy-2,5-dimethyl-3(2H)-furan-3-one	ACAL-APR-37-00	Value: 2.341% Retention time: 9.994 min
2,5-Dimethyl-4-hydroxy-3(2H)-furanone	ACAL-APR-37-00	Value: 0.853% Retention time: 11.242 min
2-[(Dimethylamino)Methyl]-4-Methoxyphenol	ACAL-APR-37-00	Value: 0.041% Retention time: 36.121 min
2-[2-Hydroxyethyl]-9-[beta-d-ribofuranosyl]hyp oxanthine	ACAL-APR-37-00	Value: 0.187% Retention time: 7.419 min
2-Acetyl-2-hydroxy-gamma-butyrolactone	ACAL-APR-37-00	Value: 1.094% Retention time: 12.323 min
2-Amino-9-(3,4-Dihydroxy-5-Hydroxymethyl-Te trahydro-Furan-2-Yl)-1,9-Dihydro-Purin-6-One	ACAL-APR-37-00	Value: 1.773% Retention time: 10.363 min
2-Amino-9-(3,4-Dihydroxy-5-Hydroxymethyl-Te trahydro-Furan-2-Yl)-3,9-Dihydro-Purin-6-One	ACAL-APR-37-00	Value: 0.184% Retention time: 18.073 min
2-Fluoro-5-[1-hydroxy-2-(methylamino)ethyl] phenol	ACAL-APR-37-00	Value: 0.045% Retention time: 38.249 min
2-Pyrimidiylamine	ACAL-APR-37-00	Value: 0.623% Retention time: 11.695 min
3,5-Dihydroxy-6-methyl-2,3-dihydro-4H-pyran-4-one	ACAL-APR-37-00	Value: 3.841% Retention time: 12.433 min
3-Deoxy-d-mannonic acid	ACAL-APR-37-00	Value: 0.187% Retention time: 22.576 min
4-(2-Amino-1-hydroxypropyl)phenol	ACAL-APR-37-00	Value: 0.096% Retention time: 23.3 min
4-(2-Aminopropyl)-Phenol	ACAL-APR-37-00	Value: 0.020% Retention time: 40.144 min
4-(2-Aminopropyl)-Phenol	ACAL-APR-37-00	Value: 0.027% Retention time: 35.862 min
4-[1-Hydroxy-2-(methylamino)ethyl]-1,2-Benze nediol	ACAL-APR-37-00	Value: 0.043% Retention time:15.725 min
4-Mercaptophenol	ACAL-APR-37-00	Value: 6.725% Retention time: 13.287 min
5-O-hexyl-d-Galactitol	ACAL-APR-37-00	Value: 0.059% Retention time: 24.18 min
6-Fluoro-4-hydroxy-2-methylquinoline	ACAL-APR-37-00	Value: 0.047% Retention time: 37.945 min
8-[(2-furanylmethyl)amino]-3,9-dihydro-1,3-dim ethyl-1H-Purine-2,6-dione	ACAL-APR-37-00	Value: 0.218% Retention time: 37.298 min
Acetic Acid, 2-Methylpropyl Ester	ACAL-APR-37-00	Value: 0.170% Retention time: 19.988 min
Butanoic Acid, 3-Oxo-, Ethyl Ester	ACAL-APR-37-00	Value: 0.308% Retention time: 8.092 min
Carbamic acid, (2-chloroethylidene)bis-, diethyl ester	ACAL-APR-37-00	Value: 0.197% Retention time: 12.012 min
Crinan-1,3-Diol	ACAL-APR-37-00	Value: 0.416% Retention time: 40.015 min
Crinan-1-Ol	ACAL-APR-37-00	Value: 0.754% Retention time: 36.367 min
Cyanoacetylurea	ACAL-APR-37-00	Value: 0.072% Retention time: 21.793 min
delta-Elemene	ACAL-APR-37-00	Value: 1.232% Retention time: 14.839 min
d-Glycero-d-galacto-heptose	ACAL-APR-37-00	Value: 0.464% Retention time: 13.002 min
Diethylalpha-acetylglutarate	ACAL-APR-37-00	Value :1.028% Retention time: 13.675 min
Elemene	ACAL-APR-37-00	Value: 0.277% Retention time: 15.835 min
Erythritol	ACAL-APR-37-00	Value: 7.221% Retention time: 12.795 min
Furfuralcohol	ACAL-APR-37-00	Value: 1.470% Retention time: 7.905 min
Gamma-Sitosterol	ACAL-APR-37-00	Value: 0.368% Retention time: 49.213 min
dl-Glyceraldehyde	ACAL-APR-37-00	Value: 1.225% Retention time: 10.279 min
Glycolic Acid	ACAL-APR-37-00	Value: 7.184% Retention time: 7.601 min
Glycolic Acid, Ethyl Ester	ACAL-APR-37-00	Value: 3.278% Retention time: 6.753 min
Iso-Caryophyllene	ACAL-APR-37-00	Value: 0.476% Retention time: 33.029 min
Isopropyl Alcohol	ACAL-APR-37-00	Value: 0.843% Retention time: 7.251 min
Kaur-16-En-18-Oic Acid	ACAL-APR-37-00	Value: 0.066% Retention time: 35.054 min
Lupetazine	ACAL-APR-37-00	Value: 1.314% Retention time: 10.997 min
Methyl ester of 3-hydroxy-4-methyl-pentanoic acid	ACAL-APR-37-00	Value: 2.947% Retention time: 13.519 min
Methyl ester of 3-hydroxy-4-methyl-pentanoic acid	ACAL-APR-37-00	Value: 2.947% Retention time: 13.519 min
Minusine	ACAL-APR-37-00	Value: 0.002% Retention time: 35.565 min
*N*-(2-Aminopropanoyl)(methyl)homocysteine	ACAL-APR-37-00	Value: 0.012% Retention time: 35.325 min
*N*-(2-Methoxycarbonylethylidene)-*N*’-Dimethylh ydrazine	ACAL-APR-37-00	Value: 0.681% Retention time: 14.322 min
*N*-(3,5-Dinitropyridin-2-yl)-l-aspartic acid	ACAL-APR-37-00	Value: 0.040% Retention time: 35.422 min
*N*-2,4-Dnp-l-arginine	ACAL-APR-37-00	Value: 0.046% Retention time: 36.496 min
N-Acetyl-d-serine	ACAL-APR-37-00	Value: 0.082% Retention time: 12.09 min
Oxalic acid, dicyclobutyl ester	ACAL-APR-37-00	Value: 2.476% Retention time: 6.352 min
Palmitic acid	ACAL-APR-37-00	Value: 0.081% Retention time: 26.845 min
Pentadeuterio-2-Acetyl-1-Pyrroline	ACAL-APR-37-00	Value: 0.018% Retention time: 15.557 min
1, 4-Piperazine	ACAL-APR-37-00	Value: 0.065% Retention time: 15.137 min
Pregn-5-Ene-3,20-Diamine	ACAL-APR-37-00	Value: 0.133% Retention time: 36.638 min
Propanoic acid	ACAL-APR-37-00	Value: 0.582% Retention time: 10.634 min
Protoanemonine	ACAL-APR-37-00	Value: 1.111% Retention time: 8.429 min
5-Methyl-4,6-pyrimidinediol	ACAL-APR-37-00	Value: 0.777% Retention time: 11.534 min
Pyruvic Acid methyl ester	ACAL-APR-37-00	Value: 2.089% Retention time: 6.831 min
Tetraacetyl-d-xylonic nitrile	ACAL-APR-37-00	Value: 0.014% Retention time: 23.876 min
Urethylane	ACAL-APR-37-00	Value: 10.967% Retention time: 7.122 min
Xanthosine	ACAL-APR-37-00	Value:1.365% Retention time: 17.925 min

### 3.2 Morphological and Characterization of AD-MSCs

AD-MSCs displayed a rounded and suspended shape on the first day of culture, Figure 2A1,A2. By the second day of development, the cells transformed into thin, linked spindles, Figure 2A3. After 7 days, some AD-MSCs in passage 1 (P1) exhibited a spindly morphology, Figure 2B1,B2. At 10 days, certain cells in passage 2 (P2) formed small colonies, Figure 2C1,C2. Some cells acquired fibroblastic properties in passage 3 (P3) at 14 days, Figure 2D1,D2. Immunocytochemistry results showed positive localization of CD105 (Figure 3A1,A2) and CD90 (Figure 3B1,B2) in the AD-MSC cytoplasm, while CD45 showed a negative reaction, Figure 3C,C2.

**Figure 2:**
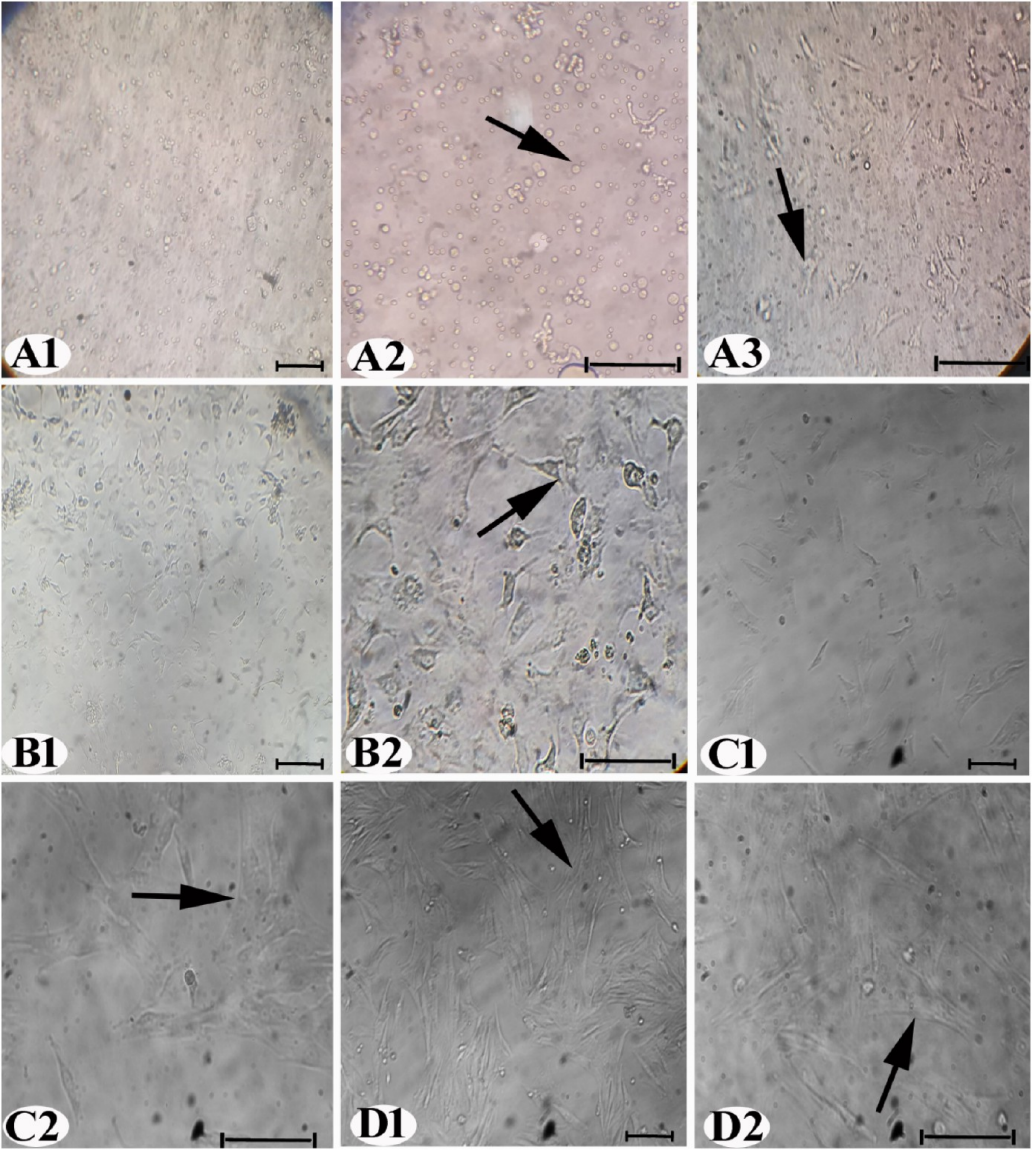
(A–D) Inverted microscopy identification of AD-MSCs at different passages (A1, A2, and A3), P0; (B1 and B2), P1; (C1 and C2), P2; and (D1 and D2), P3. AD-MSCs were distinguished by their morphology and growth. The AD-MSCs are visible with the arrows (200× and 400×).

**Figure 3:**
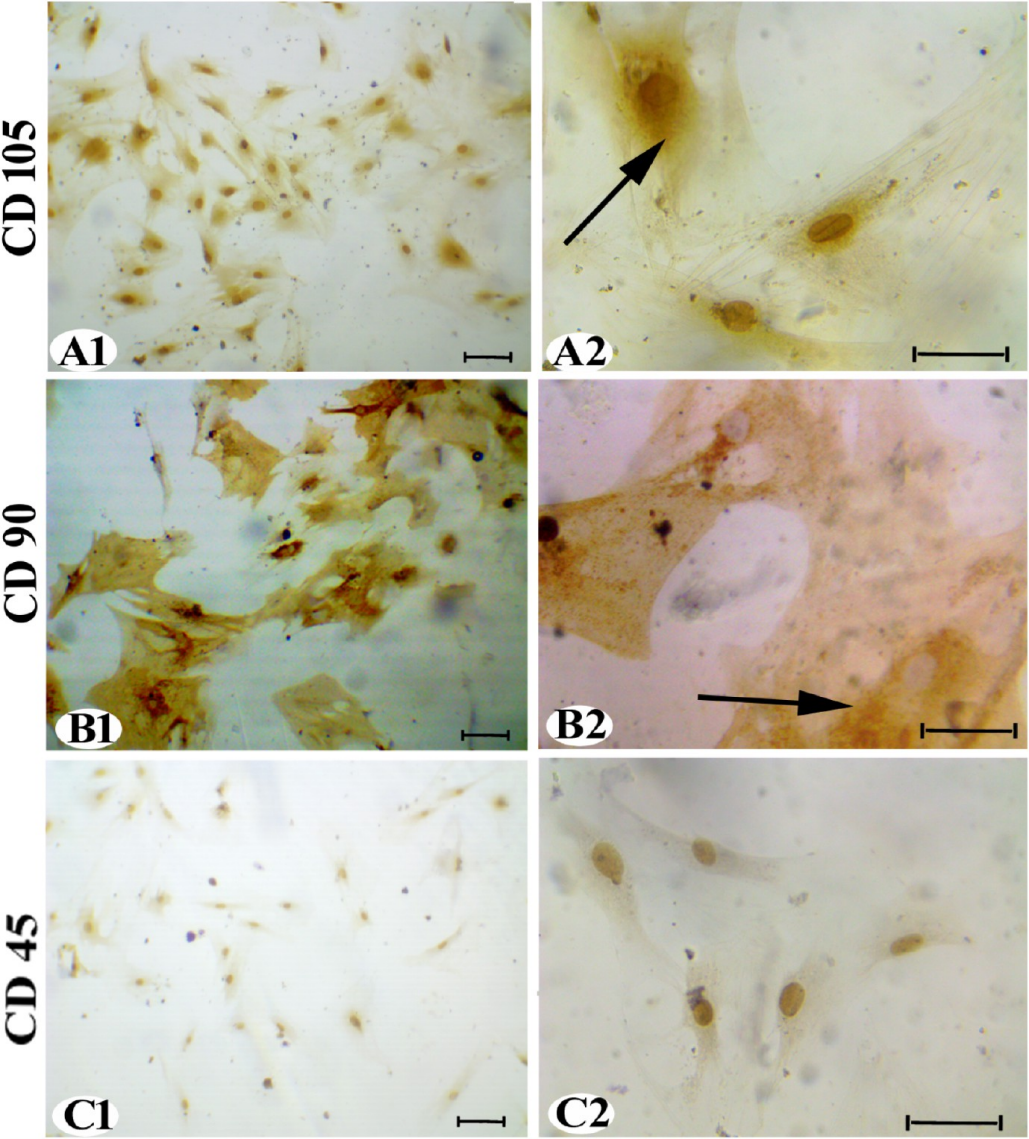
AD-MSC (P3) immunocytochemistry staining: (A1 and A2) CD105 and (B1 and B2) CD90 expressions were (+) reactions (arrow) and (C1 and C2) CD45 expression was (−) reaction (200× and 400×).

### 3.3 DNA Damage Examination

The comet images of the control groups (Figure 4A–C) showed intact nuclei in liver tissues, with rounded cells displaying no tail appearance and intact DNA. In contrast, the IQ group exhibited severely damaged DNA with dispersed tail migration, Figure 4D. However, the IQ + AD-MSCs and IQ + G groups showed more intact DNA molecules and fewer comet cell counts, Figure 4E,F, respectively.

**Figure 4:**
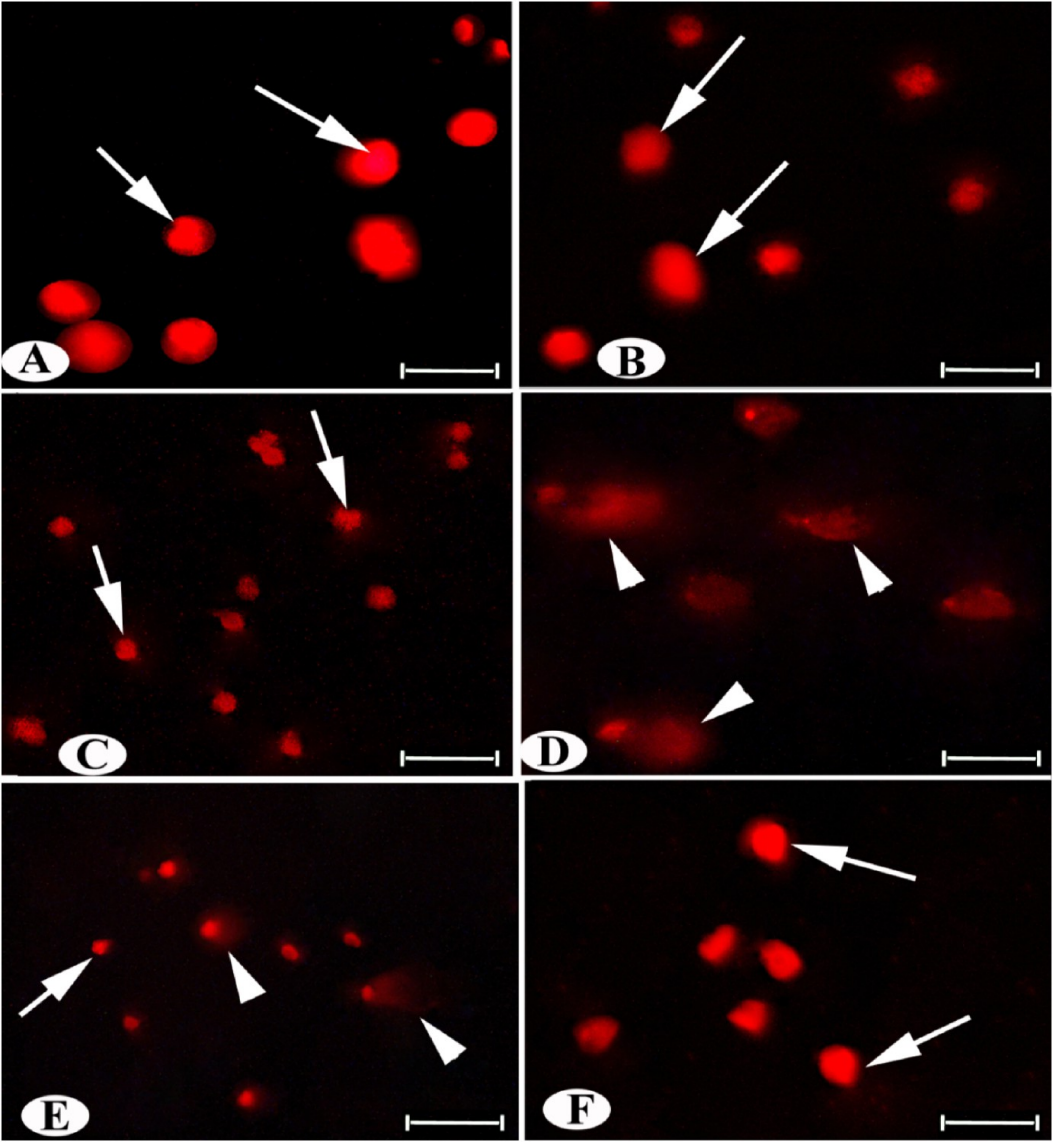
Photomicrographs of rat hepatocytes obtained with the Comet assay using a fluorescent microscope showing (A–C) normal hepatocytes with intact DNA (arrow); (D) hepatocytes of IQ intoxication with a high amount of damaged DNA (arrowhead); liver cells in IQ + AD-MSCs (E) with lower DNA damage and intact DNA than the IQ + G group (F). Scale bar: 50 μm.

Exposure to IQ for 30 days resulted in a significant increase in DNA damage in liver tissue, as evidenced by a significant increase in the mean values of the tail olive moment, tail moment, %DNA tail, and tail length (1070.15%, 3535.44%, 385.8%, and 1183.74%, respectively) compared to the control group.

Conversely, treatment with graviola and AD-MSCs indicated significantly decreased DNA damage parameters in liver tissues, with a decrease in the mean values of tail olive moment, tail moment, %DNA tail, and tail length (80.69%, 85.88%, 77.02%, and 91.49%, respectively, for graviola; 63.83%, 63.47%, 72.96%, and 84.15%, respectively, for AD-MSCs) compared to the IQ group, Figure 5.

**Figure 5:**
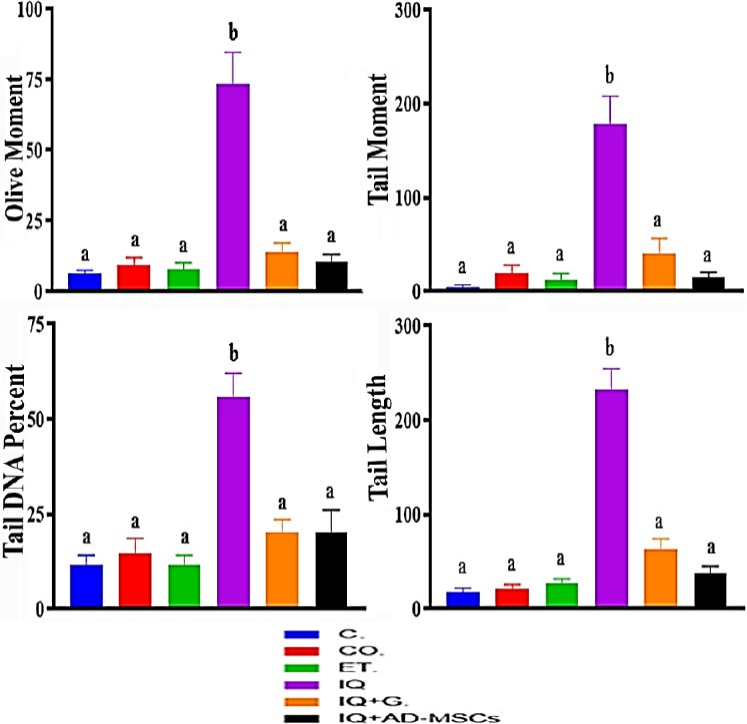
Effect of injection of AD-MSCs and oral administration of graviola on Comet assay parameters in liver tissue of rats intoxicated with IQ. Data are represented as the mean ± SE. Unlike superscript letters in the same column are significantly different at *P* < 0.05.

### 3.4 Immunohistochemistry Examination

The livers of the control male rats showed a negative p53 reaction, as shown by immunohistochemical detection, Figure 6A–C. However, most hepatocytes in male rats given IQ showed a considerable rise in fine, homogeneous brown patches of p53 protein levels, with varying degrees of localization in the cytoplasm, Figure 6D,G. In terms of statistics, the IQ group’s p53 levels were much higher (215.5%) than those of the control group.

**Figure 6:**
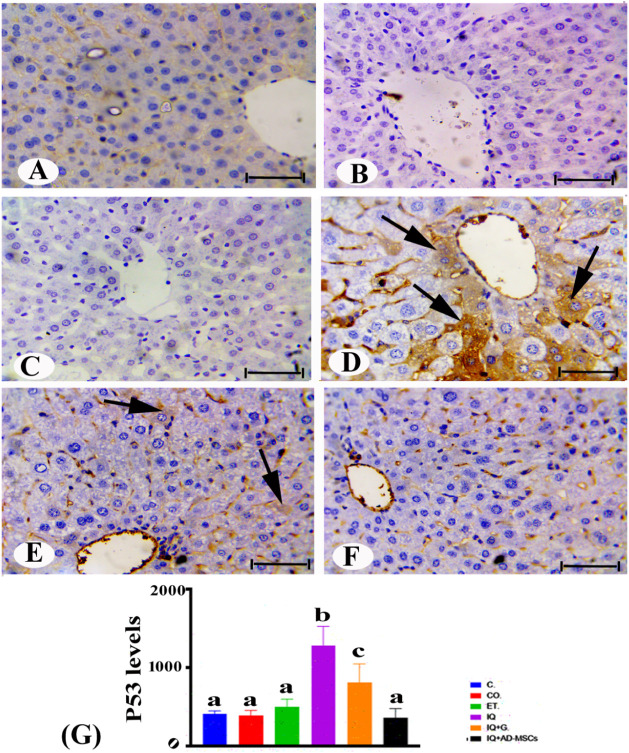
Immunohistochemistry photomicrographs of liver sections of rats in different groups of the experiment showing (A–C) control group showing negative reaction of p53 expression; (D) positive reaction of p53 (arrows) in the IQ-treated group; (E and F) the IQ + G and IQ + AD-MSC groups showed moderate positive and negative immunoreactivity of p53. Scale bar: 50 μm. (G) Statistically, the values in the column with unlike superscript letters were significantly different (*p* < 0.001).

Furthermore, the IQ + G, Figure 6E,G, and IQ + AD-MSC, Figure 6F,G, groups showed a remarkable moderate and sharp decrease, respectively, in brown patches of p53 protein levels compared with the IQ group. According to one-way ANOVA, the AD-MSC treatment group significantly inhibited p53 protein levels (72.18%) more than the graviola treatment group (36.93%) compared to IQ-induced toxicity.

### 3.5 Apoptosis Detection

The occurrence of entirely green and homogeneous nuclei in Figure 7A–C suggests that the control groups had many normal cells and few cell deaths. In comparison to the control group, the effects of IQ exposure showed a considerable rise in apoptosis and a decrease in normal cells (227.5% and 68.22%, respectively). Pyknotic nuclei, fragmented nuclei with abundant brilliant green chromatin, nuclear destruction, and nuclear fading were among the morphonuclear alterations seen, indicating severe apoptosis in the IQ group, Figure 7D,G.

**Figure 7:**
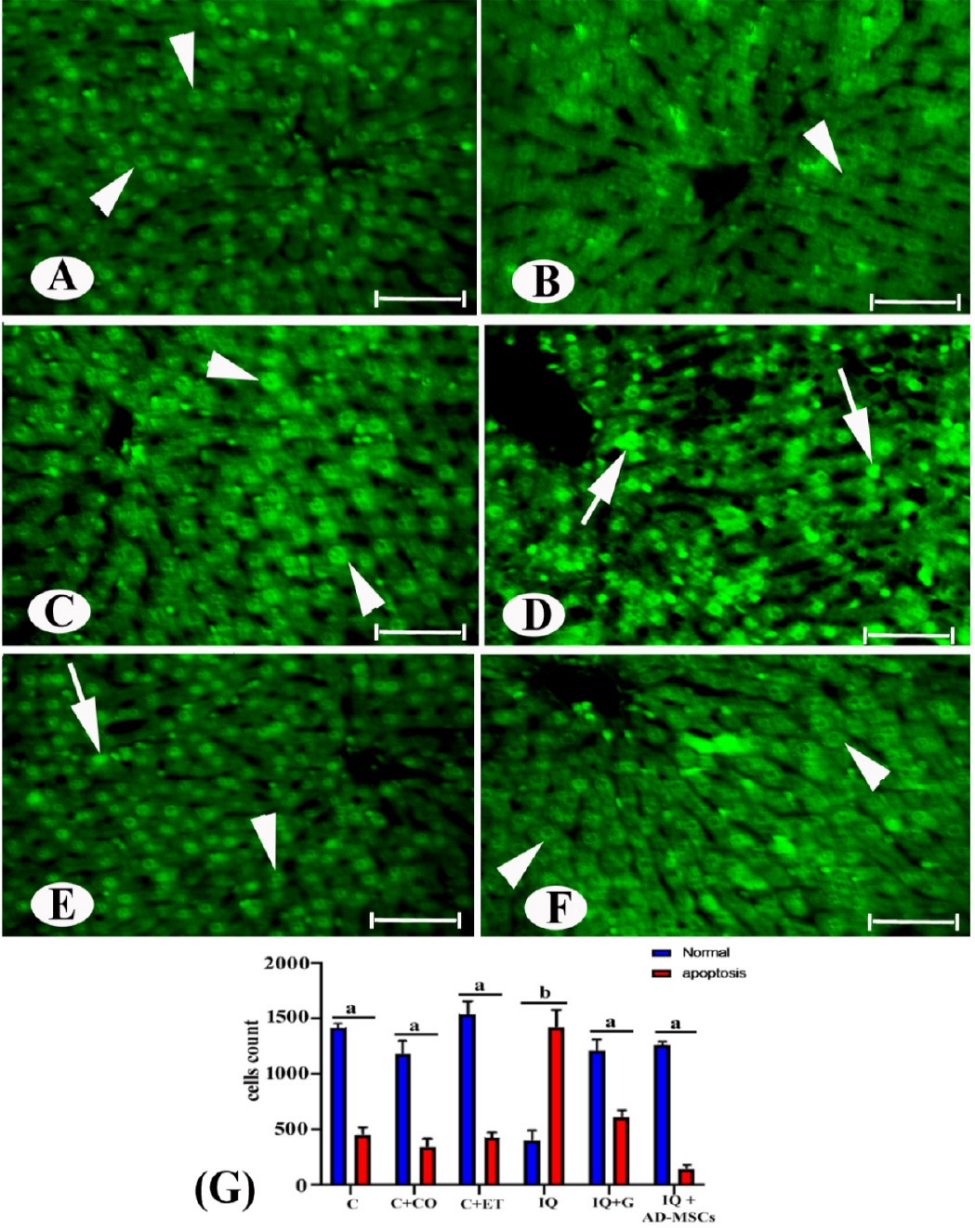
Photomicrographs of liver sections stained by AO fluorescent dyes in the different experimental groups showing (A–C) control with normal cells (arrowhead); (D) IQ with decreases in normal cells and increases in apoptosis (arrow); (E) IQ + G; and (F) IQ + AD-MSCs appeared nearly like control. Scale bar: 50 μm. (G) Statistically, the values in the column with unlike superscript letters were significantly different (*p* < 0.001).

In contrast, the IQ + G and IQ + AD-MSC groups showed normal green-colored nuclei appearances in stained cells, Figure 7E–G. Notably, there were significant increases and decreases in normal and apoptotic cells, respectively, in the graviola (54.40% increase in normal cells and 156.42% decrease in apoptotic cells) and AD-MSC (86.80% increase in normal cells and 171.31% decrease in apoptotic cells) groups compared to the IQ group.

### 3.6 Liver Function and Lipid Peroxidation Analysis

The current work showed that AST and ALT levels in male rats treated with IQ decreased nonsignificantly and increased significantly (16.08% and 42.97%, respectively) compared with the controls. The response of the IQ rats to graviola and AD-MSCs after 15 days of exposure resulted in a nonsignificant increase and a significant decrease in the levels of AST and ALT (10.43%, 40.86%, and 8.15%, 21.43%, respectively) compared to the IQ group, Figure 8A,B.

**Figure 8:**
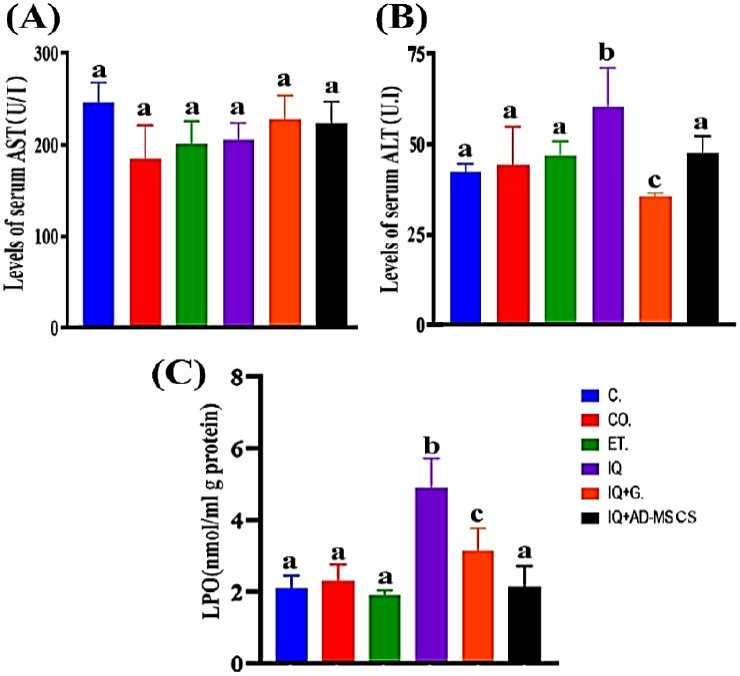
(A) The serum AST, (B) ALT, and (C) LPO in different experimental groups. Data are represented as the mean ± SE. Unlike letters in the same column are significantly different at *P* < 0.05.

Oral exposure of male rats to IQ for 30 days led to a significant increase in the level of LPO (131.2%) compared with control rats. Additionally, the IQ + G and IQ + AD-MSC groups exhibited a significant decrease in the level of LPO (36.03% and 56.04%, respectively) compared to the IQ group, Figure 8C.

### 3.7 Histological Examination

Liver sections of the control group stained by hematoxylin and eosin revealed a normal structure, Figure 9A. Liver sections from the IQ group were markedly deteriorated, Figure 9B1,B2. There was dilation and congestion in the blood sinusoids and central veins. Features of necrosis in hepatocytes, such as pyknotic nuclei and karyolysis, were observed. Most hepatocytes exhibited vacuolated cytoplasm, fatty deposition, inflammatory cell infiltration, and necrotic areas in the hepatic tissue.

**Figure 9:**
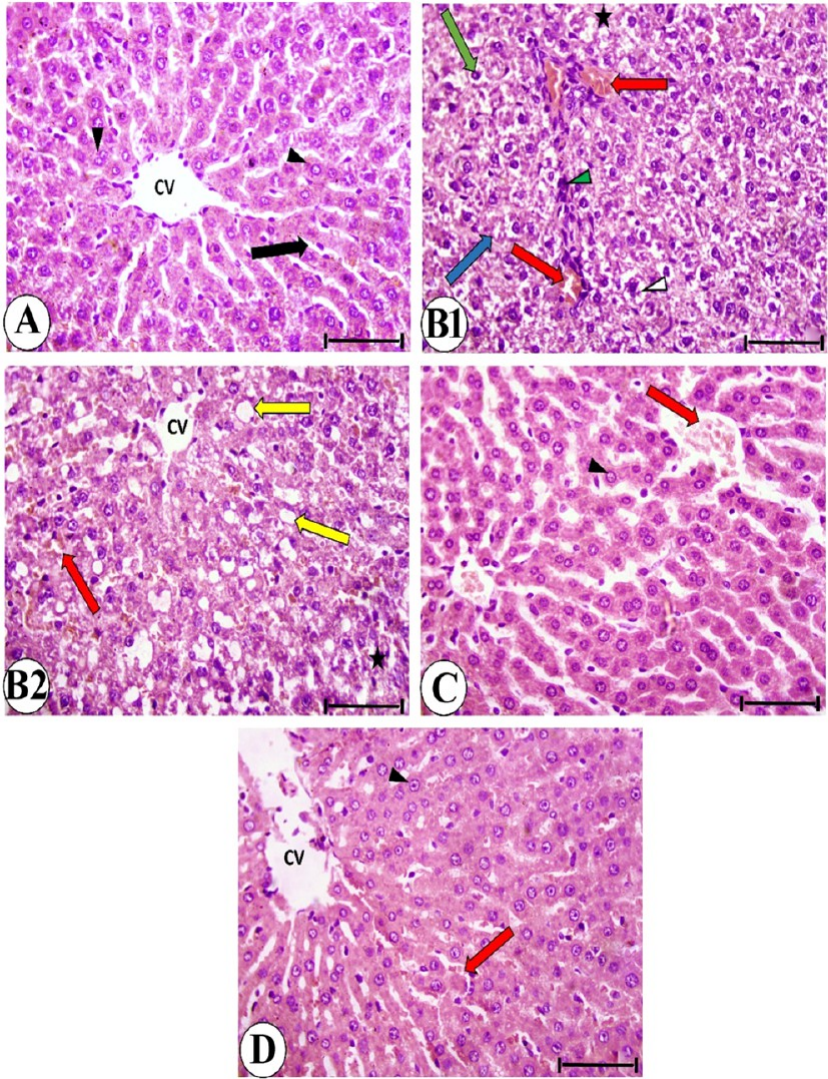
(A) Control group, (B1 and B2) IQ group, (C) IQ + G group, and (D) IQ + AD-MSC group. CV: central vein; black arrowhead: rounded vesicular nucleus; black arrow: blood sinusoid; white arrowhead: vacuolated cytoplasm; green arrow: pyknotic nucleus; blue arrow: karyolysis; asterisk: netic area; red arrow: congested blood vessel; green arrowhead: inflammatory cell infiltration; and yellow arrow: fatty deposition.

The liver in the IQ + G and IQ + AD-MSC groups showed clear improvement, Figure 9C,D. The majority of hepatocytes had rounded vesicular nuclei and normal cytoplasm. However, blood vessel congestion and dilation remained evident. The histopathological lesions in the liver of the examined groups are scored in Table 2.

**Table 2:** Scoring of Histopathological Lesions in the Livers of the Examined Groups^a^

Groups Lesions	Control	IQ	IQ + G	IQ + AD-MSCs
Pycnotic nuclei of hepatocytes	+	+++	++	+
Karyorrhexis	-	++	+	+
Karyolysis	-	++	+	+
Vacuolated cytoplasm	+	+++	++	+
Congestion of blood vessels	+	+++	++	+
Dilated blood sinusoids	+	+++	++	+
Hemorrhage	-	++	+	-
Inflammatory cells infiltration	-	++	-	-
Fatty deposition	-	++	-	-

a (−) Absent lesion, (+) slight (<25%), (++) moderate (from 25 to 50%), and (+++) severe (>50%).

### 3.8 Picro-Sirius Red Stain Examination

Liver sections stained with Picro-Sirius red from the control group in Figure 10A showed normal collagen fibers. In the IQ group, the amount of collagen fibers significantly increased (*p* < 0.001) compared to the control group, represented by the red color, Figure 10B. In the IQ + G and IQ + AD-MSC groups, the amount of collagen fibers decreased significantly (*p* < 0.05 and *p* < 0.01, respectively) compared to the IQ group, Figure 10C,D.

**Figure 10:**
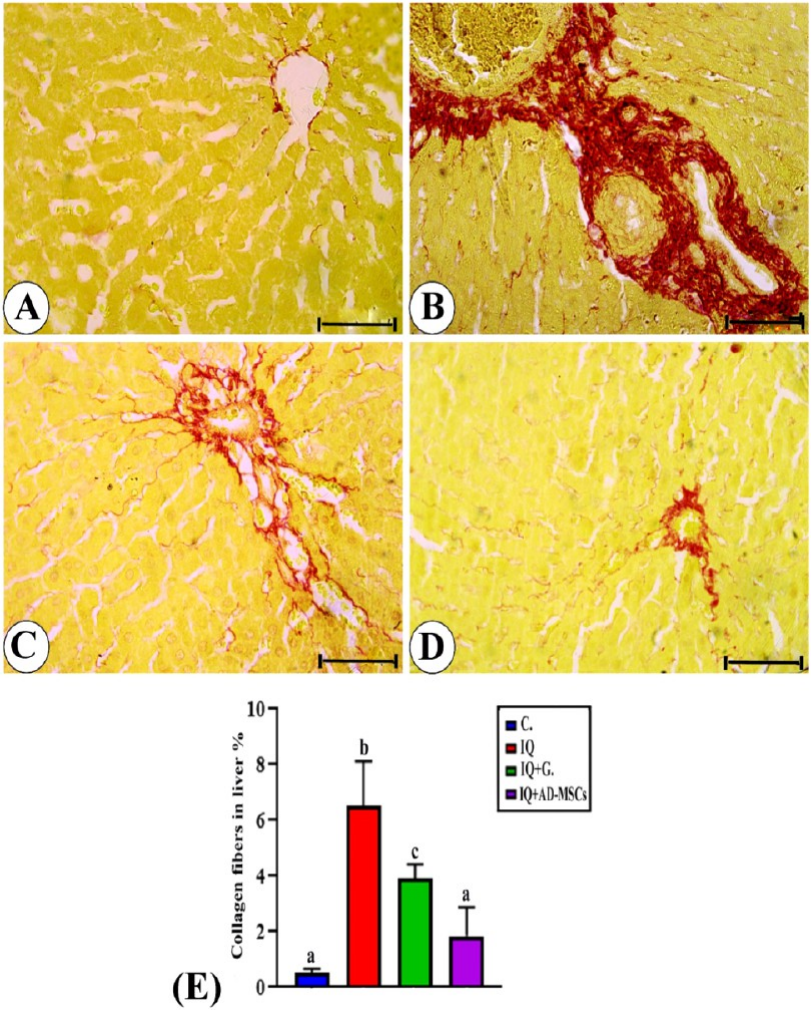
Photomicrographs of sections stained by Picro-Sirius red stain (PSS); bar = 50 μm. (A) Control group showing mild amount of collagen fibers in hepatic tissue. (B) The IQ group shows a marked increase in the amount of collagen fibers (red color). (C) The amount of collagen decreased in the IQ + G group. (D) IQ + AD-MSC group shows a nearly normal amount of collagen fibers. (E) The percentage of collagen fibers is represented as the mean ± SE. Unlike superscript letters in the same column are significantly different.

No statistical variation was observed in the collagen fibers between the control group and the IQ + AD-MSC group, but a significant difference was detected between the control group and the IQ + G group (*p* < 0.01). The percentage areas of collagen fibers in the different experimental groups are represented in Figure 10E.

## 4 Discussion

Numerous studies have documented IQ impairment and carcinogenicity, specifically targeting the liver, lung, and gut in rodents.^37^ Research has shown that IQ is linked to *p53* gene mutations in hepatocellular carcinomas, possibly caused by IQ DNA adducts in the *p53* gene.^52^ Genotoxic agents can damage DNA and result in mutations.^53,54^ In animal models, the genotoxic hepatocarcinogen 2-amino-3,8-dimethylimidazo­[4,5-f]­quinoxaline showed evidence of a practical threshold for its carcinogenicity. It forms DNA adducts at low doses, resulting in gene mutations at higher doses.^3,55^

In this study, we used an alkaline SCGE assay to investigate the impact of heterocyclic IQ-induced DNA damage in rats with varying degrees of liver injury. The results showed that the highest IQ levels were associated with the highest number of DNA strand breaks in the liver, consistent with similar findings in different tissues. The increased DNA strand breaks in the colon could result from direct DNA damage or temporary breaks caused by excision repair enzymes.^56^ MeIQ feeding for 13 days was previously shown to cause more DNA strand breakage in the colon.^56^ In cultured immortalized human liver epithelial cells examined using the Comet assay, IQ significantly increased the incidence of DNA strand breaks and the percent tail DNA in a concentration-dependent manner.^57^ Furthermore, many rodent and nonhuman primate tissues exhibit DNA adducts following IQ treatment.^58^ A substantial body of research suggests that P450s are crucial to the genotoxicity of HCAs.^59,60^

Oxidative stress is caused by an unbalanced ratio of ROS to antioxidants, resulting in cellular damage.^61,62^ Most dietary mutagens, including HCA, can covalently bind to nucleotides, forming reactive DNA adducts and causing minor nucleotide changes, leading to chromosomal abnormalities. The present study indicated that IQ can induce significant oxidative stress-induced DNA damage. However, oxidative stress seems to be an indirect contributor to DNA damage.

The human diet comprises carcinogens and substances such as antioxidants (e.g., graviola) that mitigate cancer risk.^63^ The current study’s findings highlight the role of graviola and AD-MSC in reducing IQ-induced DNA damage in rat liver tissue, which is the organ targeted by this heterocyclic aromatic amine for tumor induction. In a recent study, embryonic neural stem cell therapy showed promising results in treating spinal and brain injuries, resulting in reduced DNA damage as indicated by lower Comet assay parameters.^64^ Our study demonstrated that AD-MSC treatment significantly reduced the extent of spinal cord tissue DNA damage. Moreover, El-Shater^65^ reported that AD-MSC had a mitigating effect on lead acetate, which induces DNA fragmentation in rat brain tissue, consistent with our findings. El Makawy et al.^66^ indicated that AD-MSC’s inherent antioxidant and free radical scavenging properties are associated with its bioactive constituents.^25,67^

The hepatotoxic effect of IQ was found to be linked to an increased apoptotic pathway, as demonstrated by a significant increase in p53 expression and apoptosis following IQ exposure in the present study. Recent research has shown that IQ in zebrafish induces oxidative stress and an inflammatory response, leading to substantial liver damage.^68,69^ Consistent TUNEL tests showed that IQ increased the percentage of apoptosis, leading to cell death. Accelerated apoptosis emerged as a key toxicological mechanism of exposure to multiple toxicants, aligning with the results mentioned above.^70,71^

The current investigation indicated that graviola might mitigate liver toxicity caused by IQ-induced damage, including effects on lipid metabolism, DNA damage, and apoptosis. Graviola extracts, both aqueous and methanolic from the leaf, possess antioxidant properties and can shield DNA from H_2_O_2_ damage, as documented by George et al.^72^ and Chan et al.^73^

Gas chromatography–mass spectrometry (GC–MS) analysis confirmed the presence of phenolic components such as flavones, isoflavonols, and flavanones in the used extracts (leaf, fruit pulp, seed, and peel).^42,74^ Furthermore, the hydroxyl scavenging activity test (HRSA) revealed a robust positive correlation between the total phenolic content and the radical-scavenging abilities of each extract.^11^ Muricata extracts have demonstrated therapeutic effects, including antibacterial, antiparasitic, anti-inflammatory, angiogenic, and antitumoral properties.^75,76,77,32^ Ethanol graviola extract’s efficacy lies in inhibiting triglyceride synthesis and adipogenesis by suppressing related genes. It improves hepatosteatosis by reducing plasma fatty acid levels.^78^ Previous research has indicated that graviola extracts are abundant in rutin, kaempferol-rutinoside, anonaceous acetogenins, and vitamin U, which are representative bioactive components. These extracts act as scavengers of nitrogen and peroxyl radicals, reduce reactive oxygen species levels, and enhance immunity.^41,79^ According to our findings, AD-MSCs outperformed graviola extract in two areas: (a) reducing blood levels of liver transaminases (ALT/AST) resulting from liver damage and (b) decreasing lipid peroxidation, p53 expression, and apoptosis in liver samples, as evidenced by MDA detection. These align with earlier research, indicating p53’s involvement in IQ-induced apoptosis.^37^ This finding aligns with Sarhan’s explanation,^80^ where rats treated with a harmful substance showed significantly increased Bcl2 levels and decreased p53, caspase 3, and Bax upon graviola administration. This outcome is consistent with prior studies^81–84^ showing reduced apoptotic protein levels inhibiting cytochrome C and mitochondrial permeability. Additionally, due to its antioxidant properties, graviola reduces DNA fragmentation.

Our research showed that IQ’s cytotoxic action can damage liver cells and canaliculi, releasing enzymes into the bloodstream. This process leads to a significant increase in the liver enzymes ALT and AST, indicating the presence of a harmful substance.^85^ Furthermore, the toxicity of harmful substances induces hepatic toxicity by producing ROS. These ROS react with polyunsaturated fatty acids in cell membranes, resulting in the degradation of mitochondrial and plasma membranes and the subsequent release of hepatic enzymes.^86^ Graviola treatment preserves the structural integrity of hepatic cell membranes and aids in regenerating damaged liver cells, thanks to its antioxidant properties.^11,87^ According to the histological study, graviola inhibited the development of the hyperplastic epithelium, the physiological precursor for ductal carcinoma in situ. Additionally, it showed a reduction in DMBA-induced DNA damage.^88,89^

However, ADSC transplantation exhibited the most significant reduction compared to graviola administration, suggesting that ADSC-based therapy could improve liver function for acute liver damage.^90^ Therapeutic factors released by MSCs regulate apoptosis,^91^ inflammation,^65,92,93^ fibrosis,^94^ and angiogenesis.^95^ Previous studies have demonstrated that the high immunomodulatory effects of MSCs in autoimmune disorders are attributed to the various soluble mediators and exosomes secreted by MSCs.^96,97^ Furthermore, we observed a noticeable improvement in AST, ALT, LPO, and p53 levels after the AD-MSC treatment. These findings align with earlier research, demonstrating reduced histological alterations and pancreatic cell oxidative stress, along with enhanced antioxidant markers (GSH, CAT, and SOD).^98,99^ Compared to transplantation via the portal vein or direct liver parenchymal injection, tail vein transplantation of AD-MSCs was more effective in reducing biochemical markers such as ALT, AST, and ammonia in CCl_4_-induced liver injury.^100,101^

Additionally, we observed that MSCs exhibited potent antioxidant properties, protecting normal cells from DNA damage caused by H_2_O_2_.^65^ Numerous test results indicated that MSCs possess the ability to produce various growth factors, such as fibroblast growth factor (FGF), vascular endothelial growth factor (VEGF), keratinocyte growth factor (KGF), and others. These factors have proliferative and regenerative effects.^102,103^ Additionally, clinical investigations have shown that MSC injection has a positive therapeutic impact and is well-tolerated in the treatment of liver illnesses. Our findings indicated that using MSCs to treat rats exposed to IQ reduced their ROS levels. According to the most recent histological data, periportal hepatic necrosis associated with mononuclear cell infiltration was observed in the IQ group, while normal hepatocytes were arranged in cords around the central vein in the control and AD-MSC groups. AD-MSCs secrete various inflammatory factors, recruit inflammatory cells to the site of damage, swiftly migrate through blood vessels (see Figure S3), and express surface receptors to implant stromal cell-derived factor (SDF-1) released from the affected area.^104,105^ It has been demonstrated that AD-MSCs release higher levels of IL-6, IL-8, IL-1 receptor alpha (IL-1Rα), GM-CSF (granulocyte colony-stimulating factor), GM-CSF (granulocyte-macrophage colony-stimulating factor), and NGF (nerve growth factor) to aid in the healing of liver damage.^106,107,19^

Our data demonstrated significant reductions in inflammation and liver fibrosis following AD-MSC transplantation, a result parallel to the findings of Hao et al.^108^ As expected, AD-MSC induced hepatocyte growth factor (HGF)-mediated death in hepatic stellate cells (HSCs) to halt liver fibrosis. They similarly suppressed HSC growth and activation, as Hu et al. reported.^100^ Additionally, AD-MSCs significantly reduced the production of collagen I and III, preventing hepatic fibrogenesis by enhancing HSC death, inhibiting HSC activation, and upregulating HGF.^100,109^

Our current study utilized AD-MSCs to increase the survival rate of rats’ livers. The necrotic areas displayed enhanced liver function and improved liver regeneration while maintaining normal histology in pigs.^110,111^ Moreover, this result supports the hypothesis that MSCs play a dual role in repopulating liver cells by retaining the Kupffer cell stem cells or developing into hepatic cells.

## 5 Conclusion

The current study findings revealed that AD-MSCs and graviola treatment alleviated IQ-induced liver tissue injury, reducing DNA damage and apoptosis. Furthermore, they treated fibrosis by decreasing p53 and LPO levels and liver function proteins (ALT and AST levels). In rats, MSC therapy is the best against IQ toxicity compared to graviola oral administration, which regulates through therapeutic factors secreted by AD-MSCs and their ability to generate several lineages. Thus, AD-MSCs are a potentially effective hepatoprotective therapy, while graviola may serve as a protective and enhancement strategy for hepatic deficiencies.

## Supplementary Material



## Notes

Additional Figures S1–S3 and Table S1 as mentioned in the text and supplementary data (PDF)

## List of Abbreviations

IQ: 2-Amino-3-methylimidazo [4,5-f]­quinoline
AD-MSCs: Adipose-derived mesenchymal stem cells
G. graviola; IHC: Immunohistochemistry
AO: Acridine orange
SCGE: Single cell gel electrophoresis
ROS: Reactive oxygen species
ALT: Alanine aminotransferase
AST: Aspartate aminotransferase
LPO: Lipid peroxidation
