# Supplementary material for: Graviola Extract versus Adipose-Derived Mesenchymal Stem Cells as Therapeutics in Repairing Liver Damage Caused by 2‑Amino-3-Methylimidazo[4, 5‑f]quinoline
Source: ACS Omega. 2025 Nov 5;10(45):53848–64. doi: 10.1021/acsomega.5c03088 (PMC12631689; doi:10.1021/acsomega.5c03088)
Supplement: Supplementary file 1 [file ao5c03088_si_002.pdf]

Graviola extract versus adipose-derived mesenchymal stem cells as therapeutics in repairing liver damage caused by 2-amino-3-methylimidazo[4, 5-f]quinoline

Doaa Hamada Abd El-Hafeez Thabet[1], Mona M. Atia[1,*], Hanem S. Abdel-Tawab[1], Alshaimaa A. I. Alghriany[1]

[1]Laboratory of Molecular Cell Biology, Zoology and Entomology Department, Faculty of Science, Assiut University, 71516, Egypt

***Corresponding author:** Mona M. Atia

Telephone: +0201061996606

Address: Laboratory of Molecular Cell Biology, Department of Zoology and Entomology, Faculty of Science, Assiut University, 71516, Egypt

Email: monatia@aun.edu.eg

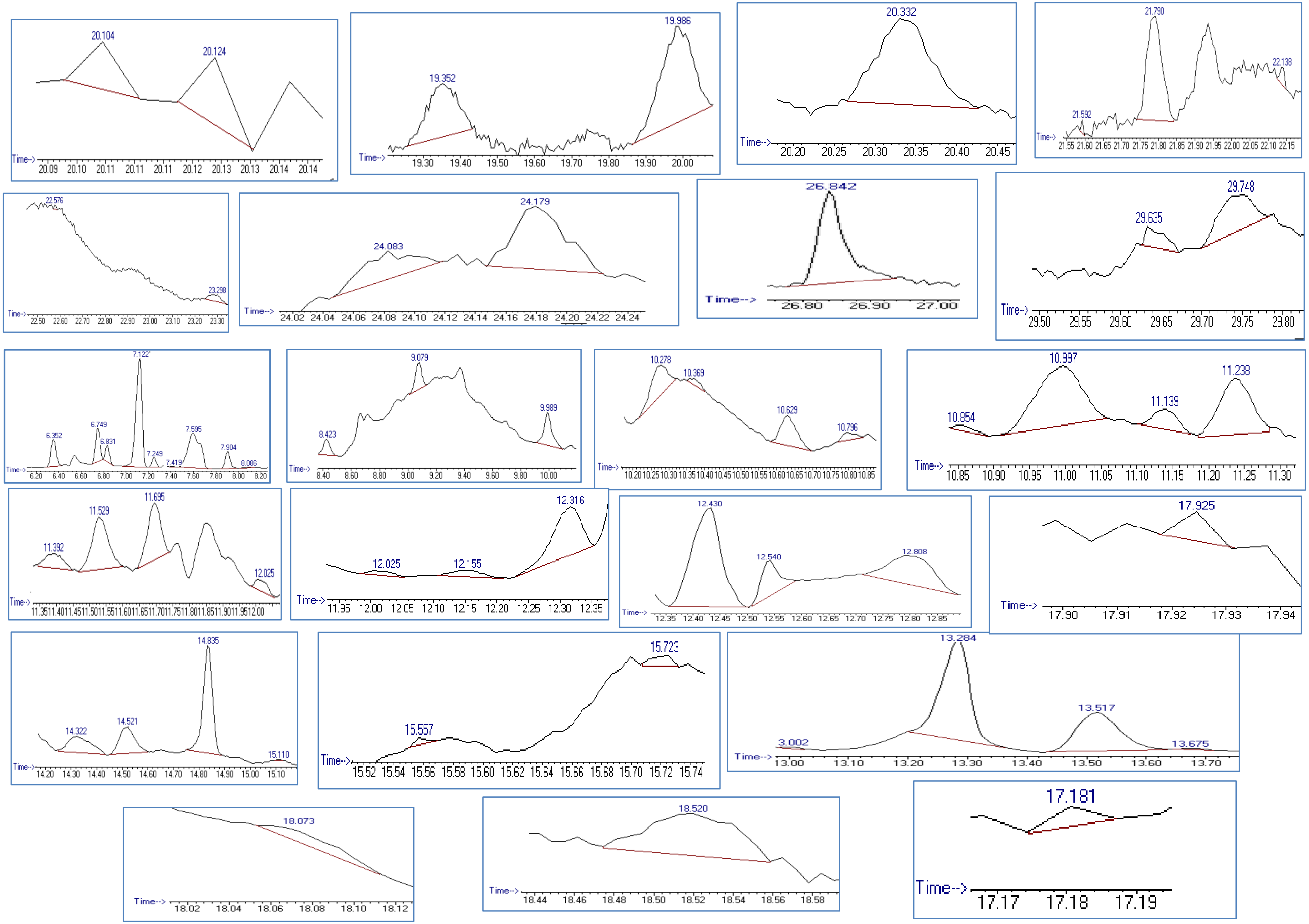

20.104
20.124
19.352
19.986
20.332
21.790
21.592
22.138
22.576
23.298
24.083
24.179
26.842
29.635
29.748
7.122
6.352
6.749
6.831
7.249
7.419
7.595
7.904
8.086
9.079
8.423
9.989
10.278
10.369
10.629
10.796
10.997
11.238
11.139
10.854
11.695
11.529
11.392
12.025
12.316
12.025
12.155
12.430
12.540
12.808
17.925
14.835
14.322
14.521
15.110
15.723
15.557
13.284
13.517
3.002
13.675
18.073
18.520
17.181
Time-->

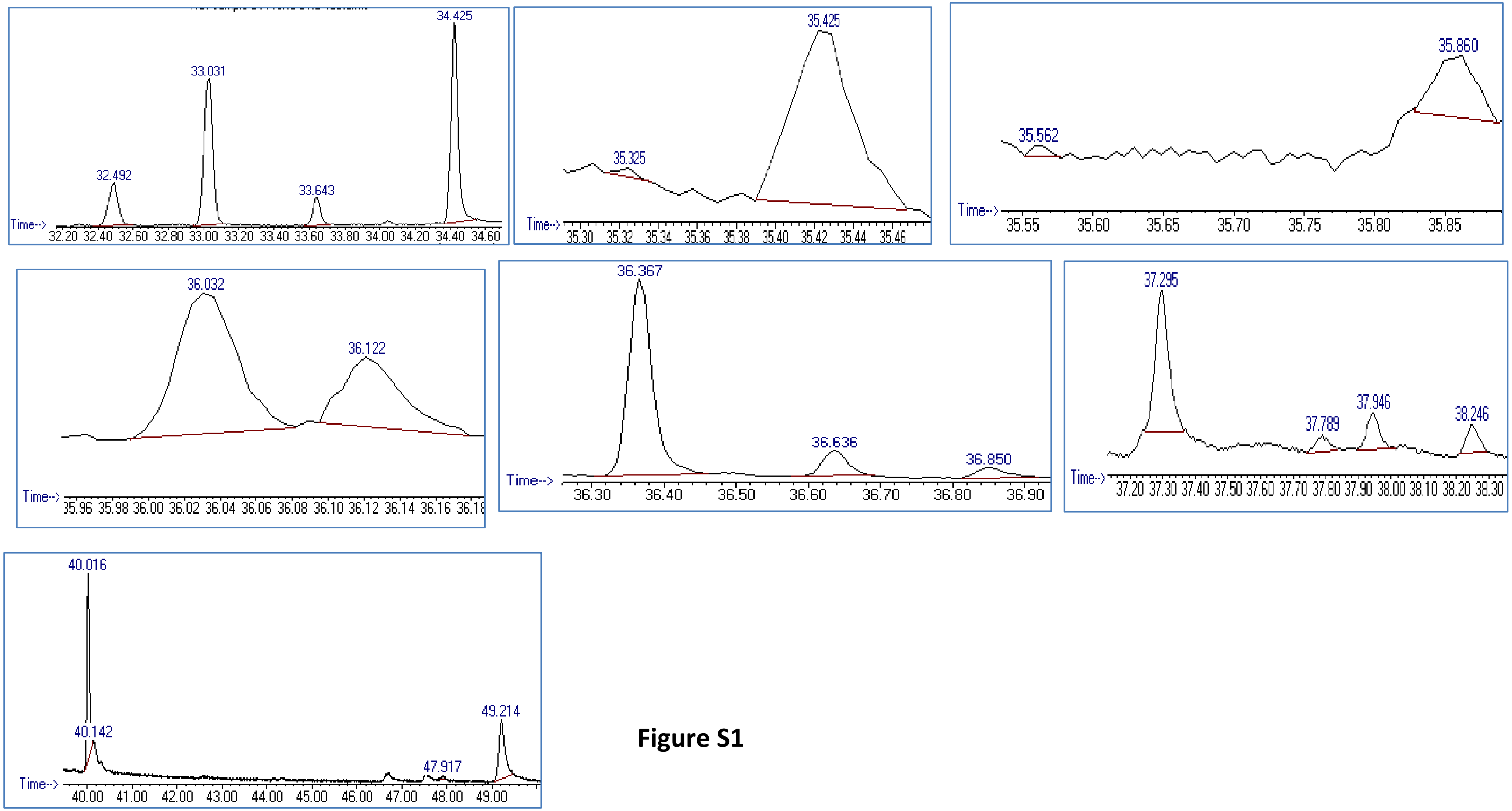
34.425
33.031
32.492
33.643
Time-->
32.20 32.40 32.60 32.80 33.00 33.20 33.40 33.60 33.80 34.00 34.20 34.40 34.60
35.425
35.325
Time-->
35.30 35.32 35.34 35.36 35.38 35.40 35.42 35.44 35.46
35.860
35.562
Time-->
35.55 35.60 35.65 35.70 35.75 35.80 35.85
36.032
36.122
Time-->
35.96 35.98 36.00 36.02 36.04 36.06 36.08 36.10 36.12 36.14 36.16 36.18
36.367
36.636
36.850
Time-->
36.30 36.40 36.50 36.60 36.70 36.80 36.90
37.295
37.946
37.789
38.246
Time-->
37.20 37.30 37.40 37.50 37.60 37.70 37.80 37.90 38.00 38.10 38.20 38.30
40.016
40.142
49.214
47.917
Time-->
40.00 41.00 42.00 43.00 44.00 45.00 46.00 47.00 48.00 49.00

Figure S1

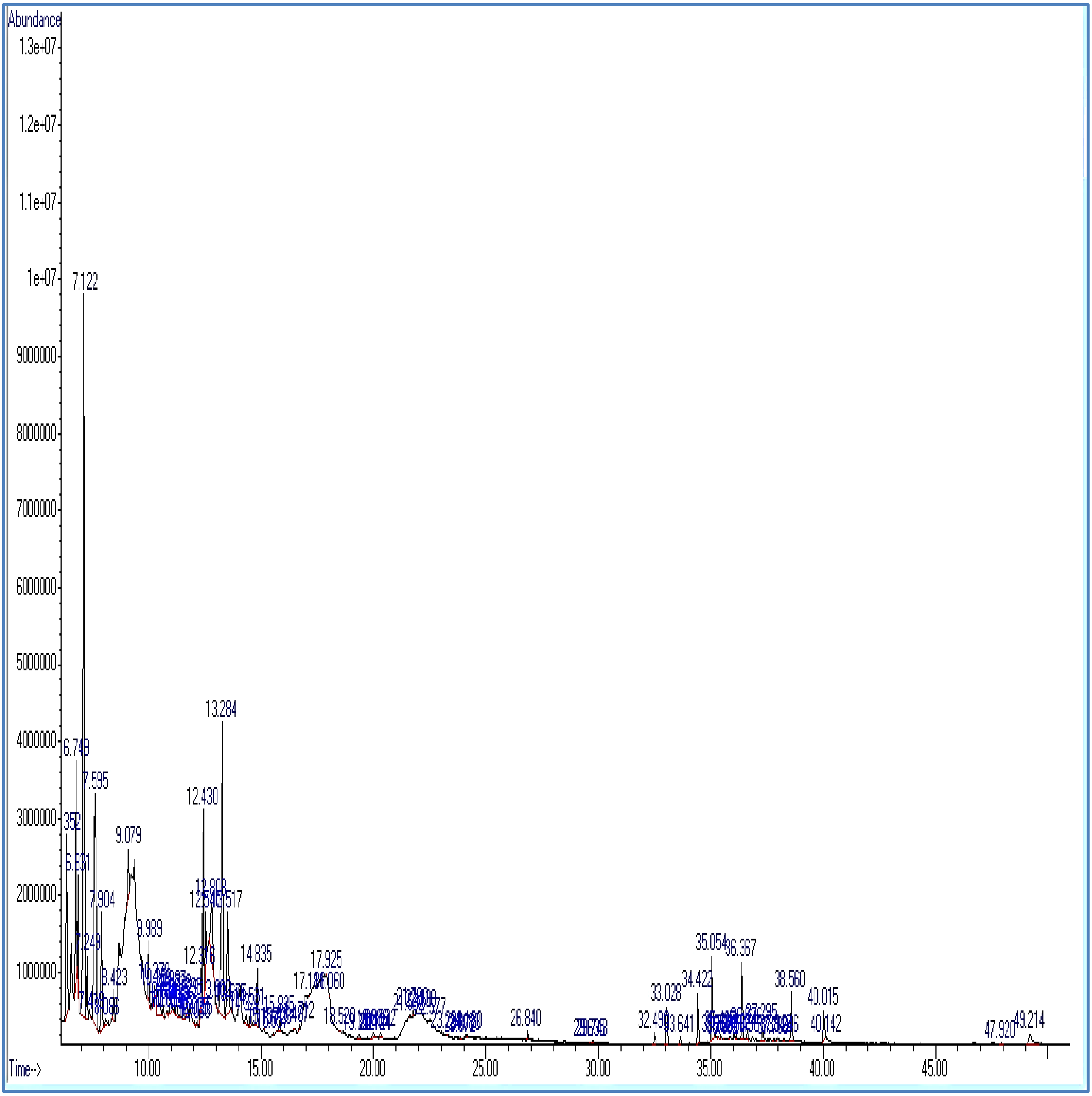
Abundance
Time-->
7.122
13.284
6.748
7.595
12.430
9.079
9.989
14.835
17.925
35.054
36.367
34.422
33.028
38.560
40.015
26.840
49.214
47.920
40.142
32.499
33.641
10.00
15.00
20.00
25.00
30.00
35.00
40.00
45.00

Figure S2

Apparatus

GC-MS (7890-5975)

Carrier gas is Hellium

Column DB-5ms (30m*0.25mm *0.25m)

Conditioning on GC/MS

Oven

Equilibration Time 0.5 min

Max Temperature 280 ^{0}C

Oven Program On

40 ^{0}C for 2 min

then 10 ^{0}C/min to 150 ^{0}C for 6 min

then 10 ^{0}C/min to 220 ^{0}C for 6 min

then 15 ^{0}C/min to 280 C for 15 min

Run Time 51 min

Run Time 48 min

2 min (Post Run) 260 ^{0}C

Mode Splitless

Flow Program 0.5 mL/min for 10.9 min

Flow then 1 mL/min per min to 1 mL/min for 30

min

MS Source 230 ^{0}C maximum 250 ^{0}C

MS Quad 150 ^{0}C maximum 200 ^{0}C

Supplementary material

Method

AD- MSCs detection method in the liver tissues

Mesenchymal therapeutic stem cells labelled with iron oxide within testicular tissue were highlighted using Prussian blue dye. After adding 50 µm iron oxide, 4 ml of RPMI medium was left for half an hour. After that, the mixture was centrifuged for 10 minutes at 2000 rpm **[Riegler et al., 2013].** Feridex-labeled MSCs were trypsinized, washed in PBS, and then reconstituted in 0.01 M PBS at a concentration of 1×1,000,000 cells/ml. The 4 µm testis slices were subjected to two PBS washes, dehydration and paraffinization, and a shaking incubation time before staining.

Following washing, the testicular sections were treated with 20% hydrochloric acid and 20% potassium ferrocyanide for 15 minutes at room temperature using Perls' reagent. After mixing the two solutions, they were shook. After being cleaned with water, the sections were counter-stained with eosin, absolute ethanol, and ethanol (90 to 70%), and then mounted with DPX **(Suvarna et al., 2019; Jasmin et al., 2012).**

Result

S3 Prussian blue staining for stem cell homing in liver tissue

Using Prussian blue stain for stem cell homing in experimental groups control, IQ, IQ+G, respectively **(Figure S3 a, b, c1 &c2)** showed negative staining. In contrast; IQ+AD-MSCs group showed, positive staining (few or more bluish pigments within the tissues) inside the liver tissue **(Figure S3 d1& d2)**.

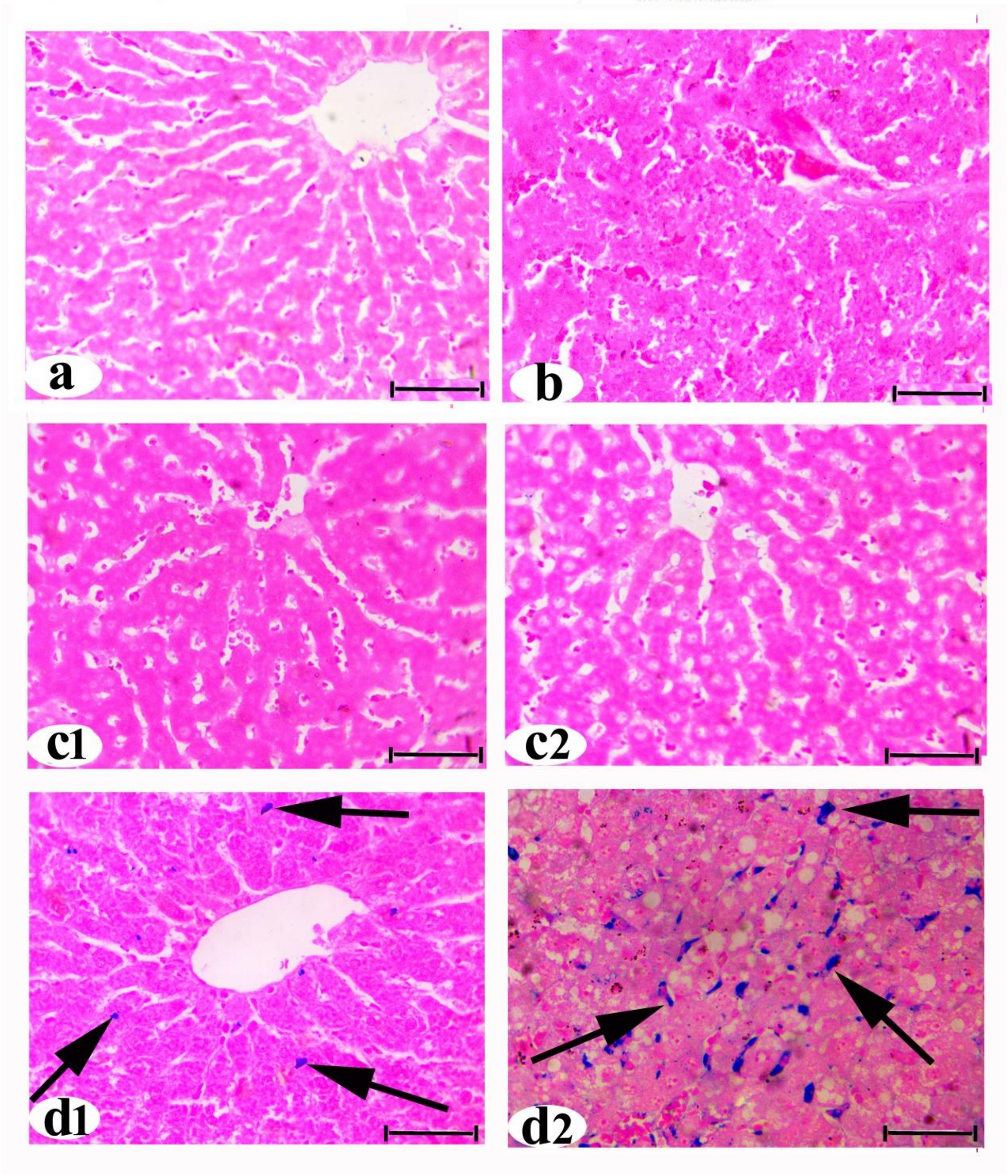

Figure S3: Photomicrograph of the liver tissue showing cells with positive reaction to Prussian blue staining (arrows). Control, IQ,and IQ + G. **(a, b , c1 , &c2)** rat with negative-stained cells, IQ+ AD-MSCs treated rats showing homing of stem cell positive-stained with Prussian blue (arrows) **(d1 &d2)** (PB stain, bar = 50 µm).

Suvarna S.K., C. Layton, J.D. Bancroft, Theory and practice of histological techniqueseighth, UK: Elsevier Health Sci (2019).

Jasmin, A.L.M. Torres, L. Jelicks, A.C.C. de Carvalho, D.C. Spray, R. Mendez-Otero, Labeling stem cells with superparamagnetic iron oxide nanoparticles: analysis of the labeling efficacy by microscopy and magnetic resonance imaging, Nanoparticles in Biology and Medicine: Methods and Protocols (2012) 239–252.

Riegler, J. A. Liew, S.O. Hynes, D. Ortega, T. O'Brien, R.M. Day, T. Richards, F. Sharif, Q.A. Pankhurst, M.F. Lythgoe, Superparamagnetic iron oxide nanoparticle targeting of MSCs in vascular injury, Biomaterials 34 (2013) 1987–1994.

Table S 1:

Analyte / Parameter	Test Method	Description
Glutaric acid, mono-phenyl ester	ACAL-APR-01-00	Value:0.168%<br>Retention time:32.492 min
1,2-Diformoxyethane	ACAL-APR-37-00	value:0.585%.<br>Retention time:10.803 min.
Carbonic Acid, Diethyl Ester	ACAL-APR-37-00	value:0.251%.<br>Retention time:10.854 min.
1-Piperidinoacetonitrile	ACAL-APR-37-00	Value:0.086%<br>Retention time:33.643 min
2-(2-Aminopropyl)phenol	ACAL-APR-37-00	Value:0.019%<br>Retention time:29.639 min
2,4-Diamino-1,3, 5-Triazin-6-One	ACAL-APR-37-00	Value:0.041%<br>Retention time:20.124 min
2-Propoxyethanol	ACAL-APR-37-00	Value:0.082%<br>Retention time:19.354 min
3-Hydroxy-2(5h)-Furanone	ACAL-APR-37-00	Value:0.152%<br>Retention time:20.337 min
2-Hydroxyethyl Thiadiazol-2-Yl Amine	ACAL-APR-37-00	Value:0.432 %<br>Retention time:21.592 min
4-Ethyl-5-[[2-[5-[(3-Ethyl-1,5-Dihydro-4-Methyl-5-Oxo-2h-Pyrrol-2-Ylidene)Methyl]-3,4-Dimethyl-2h-Pyrrol-2-Ylidene]-3,4-Dimethyl-2h-Pyrrol-5-Yl]Methylene]-1,5-Dihydro-3-Methyl-, (E,Z,Z)-2h-Pyrrol-2-One	ACAL-APR-37-00	Value:0.059%<br>Retention time:29.749 min
4-Fluoro-3-[1-hydroxy-2-(methylamino)ethyl] phenol	ACAL-APR-37-00	Value:0.017%<br>Retention time:37.79 min
5-Amino-1H-Pyrazole-4-carbothioamide	ACAL-APR-37-00	value:1.719%.<br>Retention time:12.543 min.
3-Amino-2-oxazolidone	ACAL-APR-37-00	value:0.428%.<br>Retention time:11.139 min.
2-Isopropylpiperazine	ACAL-APR-37-00	value:0.082%.<br>Retention time:12.155 min.
1,3-Dihydroxy-2-Propanone	ACAL-APR-37-00	value:5.931%.<br>Retention time:9.082 min.
(-)-Adrenaline	ACAL-APR-37-00	Value:0.043%<br>Retention time:24.083 min
1,4:3,6-dianhydro-, dinitrate D-Glucitol	ACAL-APR-37-00	value:0.530%<br>Retention time:11.398 min.
1,4-Anhydro-d-mannitol	ACAL-APR-37-00	Value:0.055%<br>Retention time:16.489 min
1,4-Diacetyl-3-acetoxymethyl-2,5-methylene-l-rhamnitol	ACAL-APR-37-00	Value:0.020%<br>Retention time:20.104 min
1,5-Bis(3,4-Dihydroxyphenyl)Pentane	ACAL-APR-37-00	Value:0.530%<br>Retention time:34.426 min
1,6-Anhydro-2,4-dideoxy-.beta.-d-ribo-hexopyranose	ACAL-APR-37-00	Value:0.162%<br>Retention time:18.52 min

12-Methylaminolauric acid	ACAL-APR-37-00	Value:0.011%<br>Retention time:47.92 min
17-(Acetyloxy)-, (4.beta.)-Kauran-18-al	ACAL-APR-37-00	Value:0.636%<br>Retention time:38.56 min
1-Butyl-2-oxo-1-propylhydrazine	ACAL-APR-37-00	Value:0.638%<br>Retention time:14.522 min
1-Deoxy-d-mannitol	ACAL-APR-37-00	Value:0.206%<br>Retention time:22.142 min
1-Nitro-2-acetamido-1,2-dideoxy-d-monnitol	ACAL-APR-37-00	Value:0.128%<br>Retention time:16.715 min
2,4-Dihydroxy-2,5-dimethyl-3(2H)-furan-3-one	ACAL-APR-37-00	value:2.341%.<br>Retention time:9.994 min.
2,5-Dimethyl-4-hydroxy-3(2H)-furanone	ACAL-APR-37-00	value:0.853%<br>Retention time:11.242 min.
2-[(Dimethylamino)Methyl]-4-Methoxyphenol	ACAL-APR-37-00	Value:0.041%<br>Retention time:36.121 min

2-[2-Hydroxyethyl]-9-[.beta.-d-ribofuranosyl]hypoxanthine	ACAL-APR-37-00	value:0.187%.<br>Retention time:7.419 min
2-Acetyl-2-hydroxy-.gamma.-butyrolactone	ACAL-APR-37-00	value:1.094%.<br>Retention time:12.323 min.
2-Amino-9-(3,4-Dihydroxy-5-Hydroxymethyl-Tetrahydro-Furan-2-Yl)-1,9-Dihydro-Purin-6-One	ACAL-APR-37-00	value:1.773%.<br>Retention time:10.363 min.
2-Amino-9-(3,4-Dihydroxy-5-Hydroxymethyl-Tetrahydro-Furan-2-Yl)-3,9-Dihydro-Purin-6-One	ACAL-APR-37-00	Value:Value:0.184%<br>Retention time:18.073 min
2-Fluoro-5-[1-hydroxy-2-(methylamino)ethyl] phenol	ACAL-APR-37-00	Value:0.045%<br>Retention time:38.249 min
2-Pyrimidiylamine	ACAL-APR-37-00	value:0.623%.<br>Retention time:11.695 min.
3,5-Dihydroxy-6-methyl-2,3-dihydro-4H-pyran-4-one	ACAL-APR-37-00	value:3.841%.<br>Retention time:12.433 min.
3-Deoxy-d-mannonic acid	ACAL-APR-37-00	Value:0.187%<br>Retention time:22.576 min
4-(2-Amino-1-hydroxypropyl)phenol	ACAL-APR-37-00	Value:0.096%<br>Retention time:23.3 min
4-(2-Aminopropyl)- Phenol	ACAL-APR-37-00	Value:0.020%<br>Retention time:40.144 min
4-(2-Aminopropyl)- Phenol	ACAL-APR-37-00	Value:0.027%<br>Retention time:35.862 min
4-[1-Hydroxy-2-(methylamino)ethyl]-1,2-Benzenediol	ACAL-APR-37-00	Value:0.043%<br>Retention time:15.725 min
4-Mercaptophenol	ACAL-APR-37-00	value:6.725%.<br>Retention time:13.287 min.
5-O-hexyl-D-Galactitol	ACAL-APR-37-00	Value:0.059%<br>Retention time:24.18 min
6-Fluoro-4-hydroxy-2-methylquinoline	ACAL-APR-37-00	Value:0.047%<br>Retention time:37.945 min

8-[(2-furanylmethyl)amino]-3,9-dihydro-1,3-dimethyl-1H-Purine-2,6-dione	ACAL-APR-37-00	Value:0.218%<br>Retention time:37.298 min
Acetic Acid, 2-Methylpropyl Ester	ACAL-APR-37-00	Value:0.170%<br>Retention time:19.988 min
Butanoic Acid, 3-Oxo-, Ethyl Ester	ACAL-APR-37-00	value:0.308%.<br>Retention time:8.092 min
Carbamic acid, (2-chloroethylidene)bis-, diethyl ester	ACAL-APR-37-00	value:0.197%<br>Retention time:12.012 min.
Crinan-1,3-Diol	ACAL-APR-37-00	Value:0.416%<br>Retention time:40.015 min
Crinan-1-Ol	ACAL-APR-37-00	Value:0.754%<br>Retention time:36.367 min
Cyanoacetylurea	ACAL-APR-37-00	Value:0.072%<br>Retention time:21.793 min
delta.-Elemene	ACAL-APR-37-00	Value:1.232%<br>Retention time:14.839 min
d-Glycero-d-galacto-heptose	ACAL-APR-37-00	value:0.464%.<br>Retention time:13.002 min.
Diethyl .alpha.-acetylglutarate	ACAL-APR-37-00	Value:1.028%<br>Retention time:13.675 min
Elemene	ACAL-APR-37-00	Value:0.277%<br>Retention time:15.835 min
Erythritol	ACAL-APR-37-00	value:7.221%.<br>Retention time:12.795 min.
Furfuralcohol	ACAL-APR-37-00	value:1.470%.<br>Retention time:7.905 min
Gamma.-Sitosterol	ACAL-APR-37-00	Value:0.368%<br>Retention time:49.213 min
dl-Glyceraldehyde	ACAL-APR-37-00	value:1.225%.<br>Retention time:10.279 min.
Glycolic Acid	ACAL-APR-37-00	value:7.184%.<br>Retention time:7.601 min

Glycolic acid, ethyl ester	ACAL-APR-37-00	value:3.278%.<br>Retention time:6.753 min.
Iso-Caryophyllene	ACAL-APR-37-00	Value:0.476%<br>Retention time:33.029 min
Isopropyl Alcohol	ACAL-APR-37-00	value:0.843%.<br>Retention time:7.251 min
Kaur-16-En-18-Oic Acid	ACAL-APR-37-00	Value:0.066%<br>Retention time:35.054 min
Lupetazine	ACAL-APR-37-00	value:1.314%.<br>Retention time:10.997 min.
methyl ester of 3-hydroxy-4-methyl-pentanoic acid	ACAL-APR-37-00	Value:2.947%<br>Retention time:13.519 min
methyl ester of 3-hydroxy-4-methyl-pentanoic acid	ACAL-APR-37-00	value:2.947%.<br>Retention time:13.519 min.
Minusine	ACAL-APR-37-00	Value:0.002%<br>Retention time:35.565 min
N-(2-Aminopropanoyl)(methyl)homocysteine	ACAL-APR-37-00	Value:0.012%<br>Retention time:35.325 min
N-(2-Methoxycarbonylethylidene)-N'-Dimethylhydrazine	ACAL-APR-37-00	Value:0.681%<br>Retention time:14.322 min

N-(3,5-Dinitropyridin-2-yl)-L-aspartic acid	ACAL-APR-37-00	Value:0.040%<br>Retention time:35.422 min
N-2,4-Dnp-L-arginine	ACAL-APR-37-00	Value:0.046%<br>Retention time:36.496 min
N-Acetyl-d-serine	ACAL-APR-37-00	value:0.082%.<br>Retention time:12.09 min.
Oxalic acid, dicyclobutyl ester	ACAL-APR-37-00	value:2.476%.<br>Retention time:6.352 min.
Palmitic acid	ACAL-APR-37-00	Value:0.081%<br>Retention time:26.845 min
Pentadeuterio-2-Acetyl-1-Pyrroline	ACAL-APR-37-00	Value:0.018%<br>Retention time:15.557 min
1, 4-Piperazine	ACAL-APR-37-00	Value:0.065%<br>Retention time:15.137 min
Pregn-5-Ene-3,20-Diamine	ACAL-APR-37-00	Value:0.133%<br>Retention time:36.638 min
Propanoic acid	ACAL-APR-37-00	value:0.582%.<br>Retention time:10.634 min.
Protoanemonine	ACAL-APR-37-00	value:1.111%.<br>Retention time:8.429 min
5-Methyl-4,6-pyrimidinediol	ACAL-APR-37-00	value:0.777%.<br>Retention time:11.534 min.
Pyruvic Acid methyl ester	ACAL-APR-37-00	value:2.089%.<br>Retention time:6.831 min.
Tetraacetyl-d-xylonic nitrile	ACAL-APR-37-00	Value:0.014%<br>Retention time:23.876 min
Urethylane	ACAL-APR-37-00	value:10.967%.<br>Retention time:7.122 min
Xanthosine	ACAL-APR-37-00	Value:1.365%<br>Retention time:17.925 min
